# Advanced Acceptor‐Substituted *S*,*N*‐Heteropentacenes for Application in Organic Solar Cells

**DOI:** 10.1002/chem.202100702

**Published:** 2021-05-29

**Authors:** Teresa Kraus, Sebastian Lucas, Pascal Wolff, Anna Aubele, Elena Mena‐Osteritz, Peter Bäuerle

**Affiliations:** ^1^ Institute of Organic Chemistry II and Advanced Materials University of Ulm Albert-Einstein-Allee 11 89081 Ulm Germany; ^2^ NVision Albert-Einstein-Allee 11 89081 Ulm Germany

**Keywords:** heteroacenes, nonfullerenic acceptors, optoelectronic properties, organic solar cells, quantum chemical calculations

## Abstract

**Ambifunctional heterpentacenes** with the heteroatom sequence SSNSS in the ladder‐type backbone were used either as donor or as nonfullerenic acceptor in solution‐processed bulk‐heterojunction solar cells. Different acceptor moieties and side chains were inserted. Synthesis and characterization of the systematically varied structural motifs provided insight in structure‐property relationships. Moreover, a dimeric heteroacene was synthesized, and the optoelectronic properties were compared to those of its monomeric counterpart.

## Graphical abstract

Functionalized *S,N*‐heteropentacenes SN5’ comprising annulated thiophene and pyrrole rings with the heteroatom sequence SSNSS in the ladder‐type backbone were developed with systematically varied structural motifs. Tunable frontier orbital energies allowed their application either as donor or as nonfullerenic acceptor in solution‐processed bulk‐heterojunction solar cells.

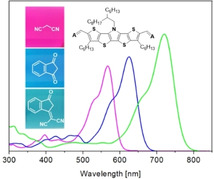

## Introduction

*S,N*‐Heteroacenes arose as important class of π‐conjugated systems and promising organic semiconductors for application in organic electronic devices such as organic field‐effect transistors or organic solar cells (OSC).^[1]^ They typically consist only of fused thiophene and pyrrole rings resulting in planar, ladder‐type structures with highly delocalized π‐electrons and can be seen as *S,N* analogues of phenacenes, which themselves represent isomeric forms of the widely investigated acenes.^[2]^ The principal synthetic strategies and methods for the construction of the fused polycyclic heteroaromatics mainly comprise S‐ and N‐heteroannulation reactions.[^3^, ^4^] In this respect, very long, structurally defined, and still soluble *S,N*‐heteroacenes with up to 13 fused thiophenes and pyrroles (SN13)^[5]^ became feasible by application of Pd‐catalyzed Buchwald‐Hartwig amination/cyclization reactions of brominated thiophene‐based precursors and amines.[^6^, ^7^] Number and sequence of the heteroatoms in the ladder‐type backbone allow for tuning of the optoelectronic properties and interesting structure‐property relationships in dependence on the conjugated chain length were deduced.[^8^, ^9^] Theoretical work on the series SN6 to hypothetical SN14 showed the linear relation of computed transition energies of electronic spectra and conjugation length of neutral, radical cationic, and dicationic species.^[10]^

**Figure 1:**
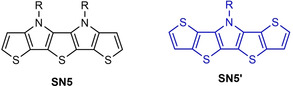
*S,N*‐Heteropentacenes **SN5** (*SNSNS*) and **SN5’** (*SSNSS*).

Attachment of alkyl side chains at various positions, in particular at the nitrogen of pyrrole rings, enhance solubility and packing in the solid state, whereas the reactive α‐positions of the terminal thiophene units in *S,N*‐heteroacenes allow for their straightforward chemical functionalization. In this respect, the attachment of terminal acceptor (A) groups at the electron‐rich *S,N*‐heteroacene donor (D) core leads to broad and strong absorption with charge‐transfer (CT) character in the visible range of the solar spectrum and well‐balanced energies of the frontier orbitals which are prerequisites for well‐functioning photovoltaic devices.^[1]^ For example, end‐capping of *S,N*‐heteroacenes SN5 (Figure 1) or SN6 with dicyanovinylene (DCV) units led to photoactive donor components, which in blends with fullerenes as acceptor reached power conversion efficiencies (PCE) of up to 7.1 % in vacuum‐ or solution‐processed bulk‐heterojunction organic solar cells (BHJ‐OSC).[^11^, ^12^, ^13^] More recently, these A‐D‐A‐type SN6 derivatives have been inverted to act as so‐called nonfullerenic acceptor (NFA) in BHJ‐OSC by attaching stronger acceptors than DCV. In this respect, the frequently used fluorinated indanone (DFIC), which causes a decrease and stabilization of both, the lowest unoccupied molecular orbital (LUMO) and the highest occupied molecular orbital (HOMO), has been implemented. By blending with donor polymer PBDB‐T power conversion efficiencies (PCE) of up to 13.2 % were achieved.[^14^, ^15^] Moreover, due to their balanced charge transport properties, SN5 and SN6 derivatives were applied as hole transport material in perovskite solar cells[^16^, ^17^] or in organic field‐effect transistors.^[18]^

In comparison to pentameric SN5 (alternating heteroatom sequence *SNSNS*), which was first synthesized by Suga et al.,^[19]^ derivative SN5’ consists of a central pyrrole core fused with thienothiophene units (*SSNSS*; Figure 1) and showed nearly identical optical properties, but a higher oxidation potential and consequently a better stabilized HOMO energy level.[^20^, ^21^]

**Figure 2:**
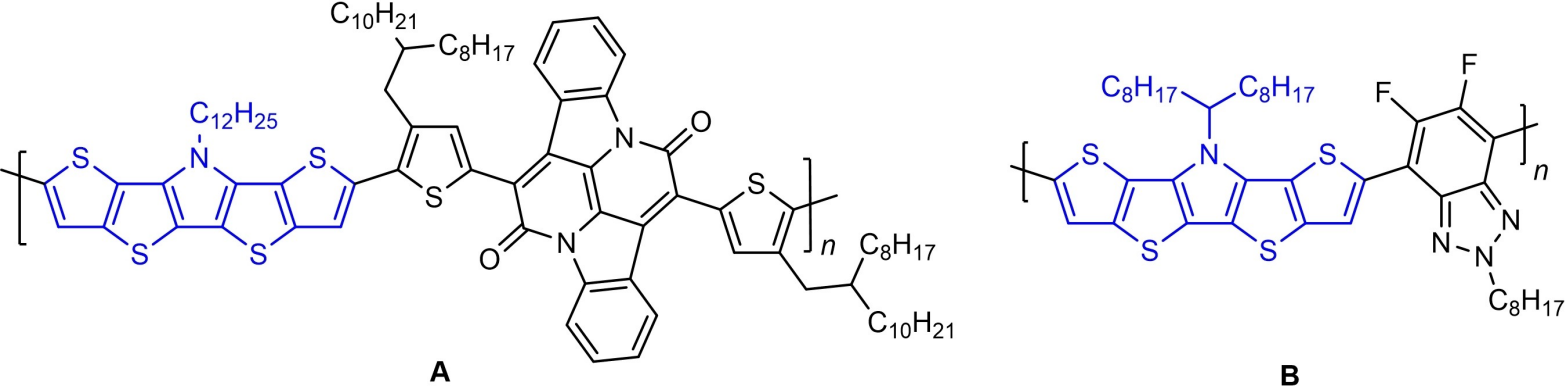
Structures of SN5’‐based D‐A copolymers **A** and **B**.

These promising electronic properties rendered SN5’ to an auspicious building block in various π‐conjugated materials for application in organic solar cells. Low bandgap D‐A copolymers **A** and **B** containing SN5’ as donor block have been implemented as photoactive layer in NIR organic photodetectors^[22]^ or in BHJ‐OSCs, respectively, resulting in 6 % power conversion efficiency (PCE) due to broad absorption, high hole mobility, and efficient exciton dissociation (Figure 2).^[23]^

**Figure 3:**
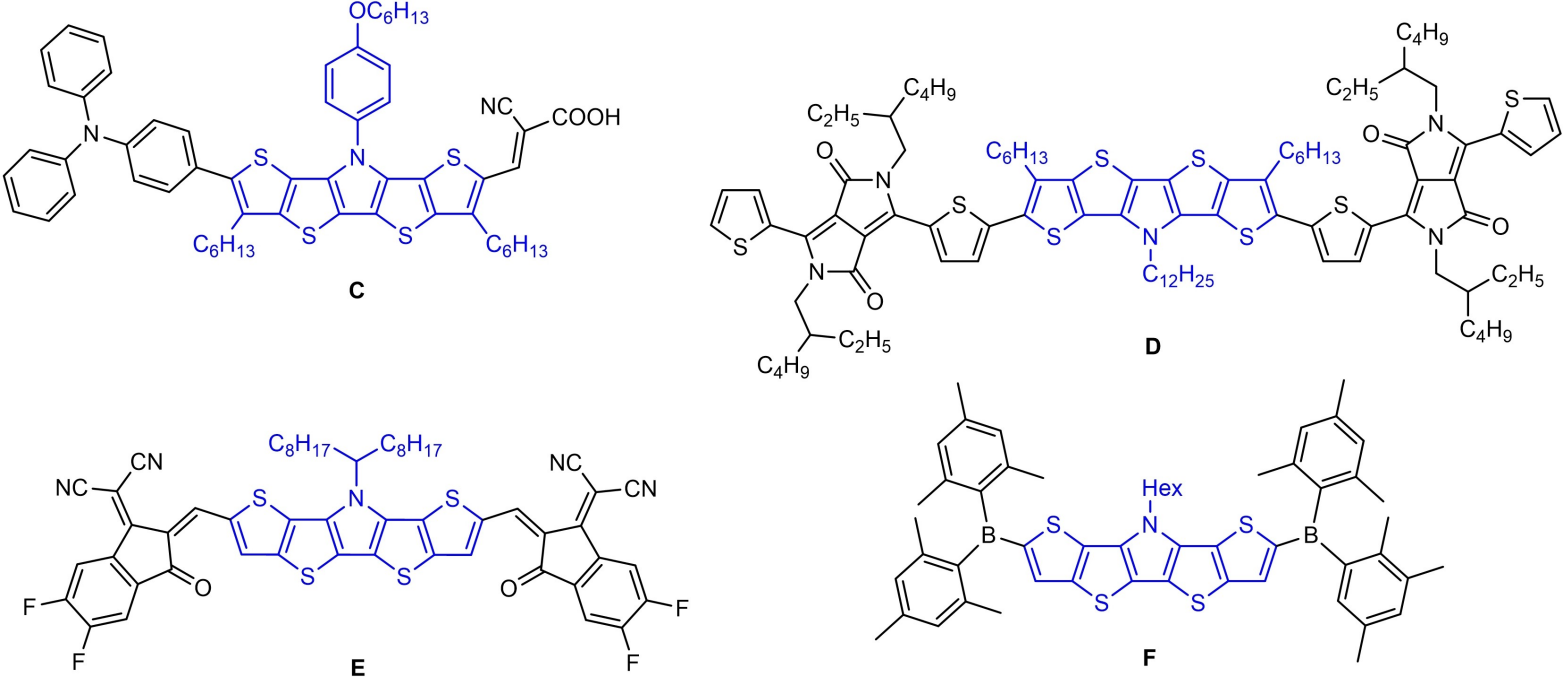
Structures of SN5’‐based co‐oligomers **C**, **D**, **E**, and **F**.

Monodisperse co‐oligomeric SN5’ derivatives of the D‐A type were synthesized for application in dye‐sensitized solar cells. The insertion of a SN5’ unit into D‐A systems such as in oligomer **C** (Figure 3) led to enhanced absorption and hence to higher short‐circuit current density and power conversion efficiency (PCE=9.4 %) in the dye‐sensitized solar cell.^[24]^ SN5’ derivatives of the A‐D‐A type, in which the electron‐rich SN5’ core is end‐capped by electron‐withdrawing units, were successfully implemented into solution‐processed BHJ‐OSCs. For example in co‐oligomeric donor **D**, the SN5’ block is connected to diketopyrrolopyrrole acceptor moieties (Figure 3) leading to a PCE of 3.8 % in BHJ‐OSCs when blended with fullerene PC_71_BM.^[25]^ The variability of the SN5’ system becomes obvious when regarding A‐D‐A system **E**, which combines SN5’ and DCIF acceptors.^[26]^ These strong acceptors substantially stabilize the frontier orbitals of the A‐D‐A co‐oligomer leading to strong NIR absorption and improved 8.9 % PCE when combined as NFA with donor polymer PBDB‐T‐2F in BHJ‐OSCs. Finally, dimesitylboron end‐capped SN5’ **F** showed a high fluorescence quantum yield allowing application as fluorescent probe in fluoride sensors.^[27]^

In continuation of our work on heteroacenes and their application in organic solar cells, we now report the further development of SN5’ derivatives with systematically varied structural motifs leading to valuable structure‐property relationships. The thereby tunable frontier orbital energies of these novel functionalized *S,N*‐heteropentacenes SN5’ allow for a rare two‐fold application: either their implementation as donor in solution‐processed BHJ‐OSCs or as NFA.

## Results and Discussion

### Synthesis of N‐alkylated A‐D‐A‐type *S,N*‐heteropentacene 7 a

**Scheme 1:**
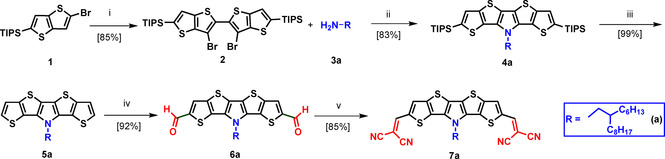
Synthesis of DCV‐functionalized SN5’ derivative **7 a**: i) *1*. LDA, THF, 0 °C; *2*. CuCl_2_, −78 °C to RT; ii) *1*. Pd(dba)_2_, dppf, NaO*t*Bu, toluene, 24 h, 110 °C; iii) TBAF⋅3 H_2_O, THF, 18 h, RT; iv) POCl_3_, DMF, DCE, 80 °C, 24 h; 2. NaHCO_3_, RT, **2 d**; v) NH_4_OAc, malononitrile, DCE, 80 °C.

The synthetic route to SN5’ derivative **7 a** is presented in Scheme 1. Triisopropylsilyl (TIPS)‐protected 2‐bromothienothiophene **1** was subjected to a halogen‐dance reaction using lithium diisopropylamide (LDA) to give isomeric 3‐bromothienothiophene,^[28]^ which is negatively charged at the α‐position. This intermediate was oxidatively homocoupled in situ in 55 % yield to dibromide **2** by the addition of copper(II) dichloride. Pd‐catalyzed ring closure with 2‐hexyldecylamine **3 a** under Buchwald‐Hartwig conditions provided TIPS‐protected pentafused SN5’ derivative **4 a** in 83 % yield. Deprotection of **4 a** with tetrabutylammonium fluoride (TBAF) gave basic SN5’ derivative **5 a** in quantitative yield. In order to prepare the A‐D‐A‐type oligomer, dialdehyde **6 a** was obtained in 92 % yield by Vilsmeier‐Haack formylation of **5 a** using large excesses of phosphoryl chloride and *N,N*‐dimethylformamide (DMF) and following hydrolysis. Then, the formyl groups were transformed into DCV moieties by treatment of dialdehyde **6 a** with malononitrile and ammonium acetate as base. Pure DCV‐SN5’ **7 a** was obtained in 85 % yield after precipitation and washing with methanol.

### Synthesis of A‐D‐A‐type *S,N*‐heteropentacenes with various acceptor moieties

**Scheme 2:**
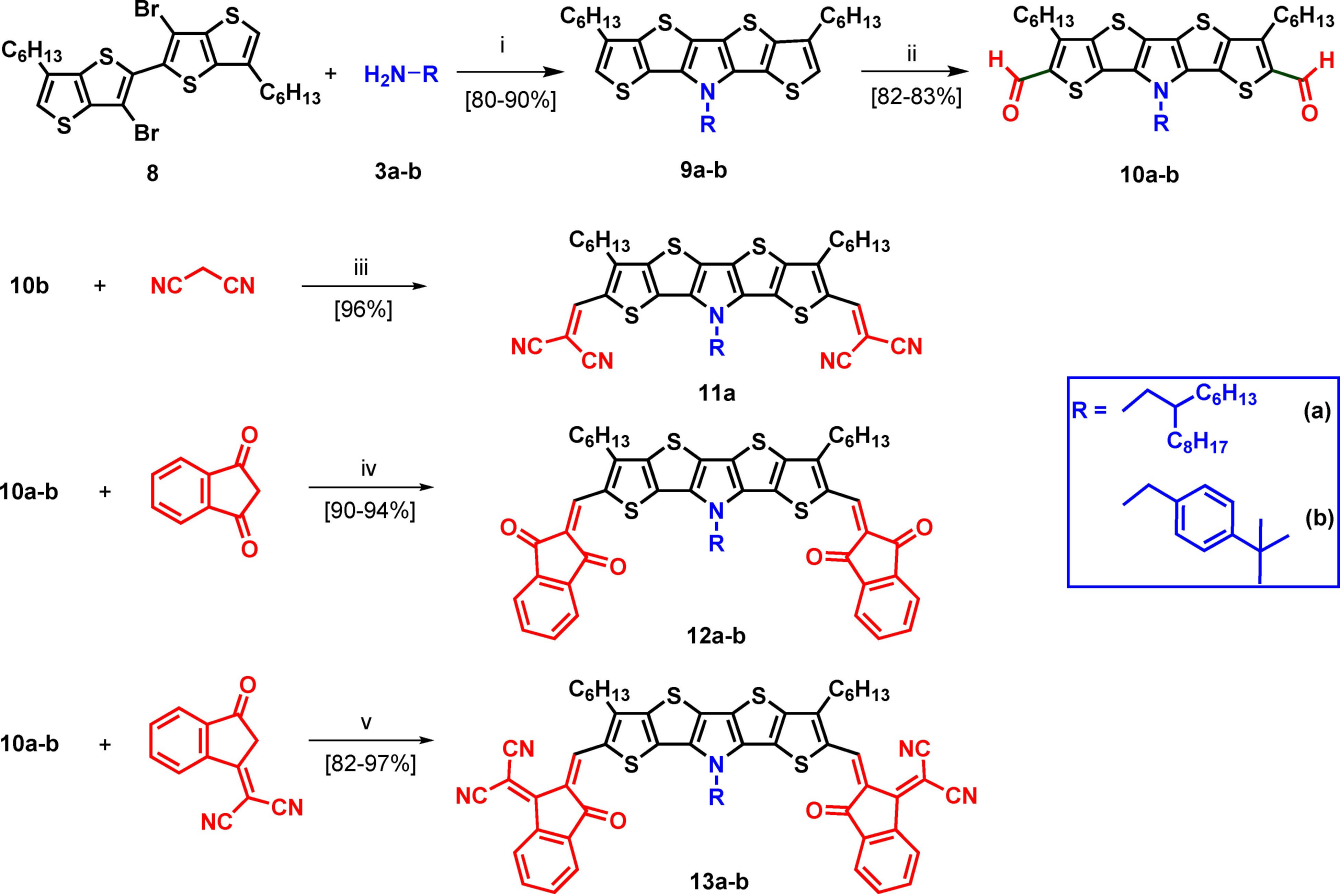
Synthesis of acceptor‐functionalized SN5’ derivatives **11 a**, **12 a**–**b**, and **13 a**–**b**: i) Pd(dba)_2_, dppf, NaO*t*Bu, 110 °C; ii) *1*. DCE, POCl_3_, DMF, 70 °C; *2*. NaHCO_3_; iii) NH_4_OAc, DCE, 80 °C, 24 h; iv) piperidine, DCE, RT, 24 h, v) DCE/ EtOH (1 : 1), 60 °C **2 d**.

In this SN5’ series, terminal acceptor blocks with varying acceptor strength were attached to the parent SN5’ core in order to investigate the influence on the optoelectronic and photovoltaic properties of the A‐D‐A system. For increased solubility, we inserted hexyl side chains at the outer β‐positions of the parent *S,N*‐heteropentacene. The synthetic route is presented in Scheme 2 and started with dibrominated dimeric thienothiophene **8**, which is structurally similar to corresponding precursor **2** and was prepared according to Lai et al.^[29]^ Ring closure to the heteropentacene was achieved by Pd‐catalyzed amination of dibromide **8** with 2‐hexyldecylamine **3 a** or 4‐*tert‐*butylbenzylamine **3 b** to result in alkylated SN5’ derivatives **9 a** and **9 b** in 80 % and 90 % yield, respectively. The latter system was prepared for comparison to corresponding SN5’ dimer **16 c** (vide infra). The extension to the A‐D‐A‐system was achieved by Vilsmeier‐Haack formylation of **9 a** and **9 b**. Resulting dialdehydes **10 a** and **10 b** (82‐83 % yield) were reacted in Knoevenagel condensation reactions with different acceptor blocks. Malononitril and ammonium acetate gave DCV derivative **11 a** in 98 % yield, 1,3‐indandione (IND) and piperidine resulted in **12 a** and **12 b** in 90–94 % yield, and dicyanomethylene‐3‐indanone (DCI) gave **13 a** and **13 b** in 82–97 % yield without base.

**Scheme 3:**
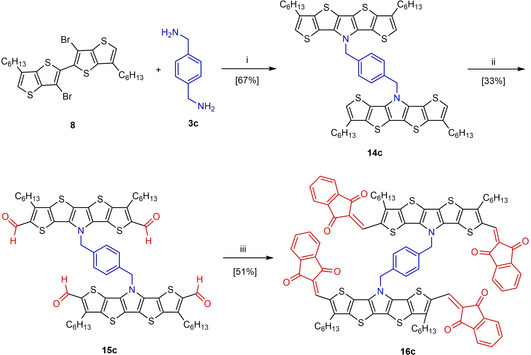
Synthesis of acceptor‐functionalized SN5’ dimer **16 c**: i) Pd(dba)_2_, dppf, NaO*t*Bu, toluene, 110 °C, **2 d**; ii) *1*. CH_2_Cl_2_, POCl_3_, DMF, 70 °C, **7 d**; *2*. NaOH, 24 h, RT; iii) 1,3‐indanone, piperidine, CH_2_Cl_2_, RT, **2 d**.

Following the same strategy, the IND‐substituted SN5’ dimer **16 c** was synthesized by employing bifunctional diamine **3 c** (Scheme 3). In this respect, fourfold Pd‐catalyzed Buchwald‐Hartwig amination of bithienothiophene **8** with *p*‐xylylene diamine **3 c** gave dimer **14 c** in 67 % yield. Subsequent Vilsmeier‐Haack formylation to tetraaldehyde **15 c** and Knoeveangel condensation with IND and piperidine provided dimer **16 c** in 51 % yield representing a coupled (A‐D‐A)_2_ system. Here, the interesting question was, how the enforced proximity of two heteroacenes would influence optoelectronic properties on one hand and the aggregation behavior on the other hand. The structures of the novel A‐D‐A SN5’ derivatives were fully characterized by NMR spectroscopy (Figures S1–S7 in the Supporting Information) and high‐resolution mass spectra (HRMS; Figures S8–S14).

### Optical and redox properties of the SN5’ derivatives

**Figure 4:**
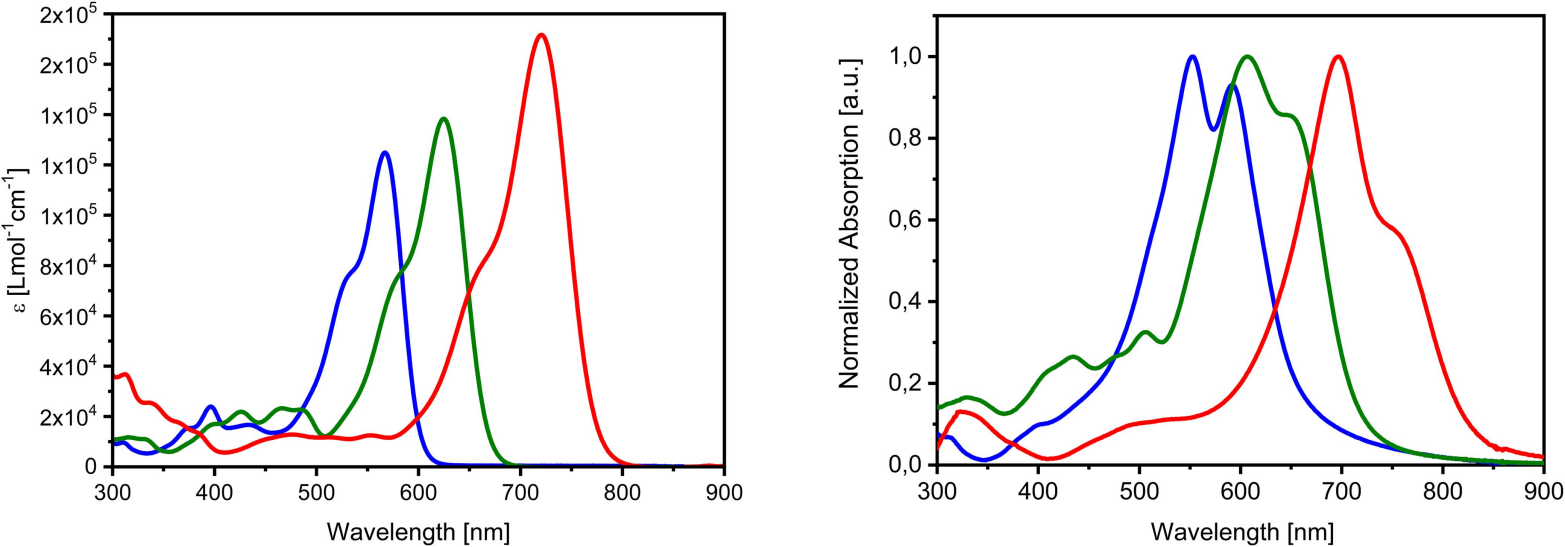
Absorption spectra of SN5’ derivatives **11 a** (blue), **12 a** (green), and **13 a** (red) recorded in CH_2_Cl_2_ (left) and in thin films doctor‐bladed from chloroform solution (right).

Spectroscopic investigations on the various SN5’ derivatives were performed by UV/vis absorption and emission spectroscopy in dichloromethane solution (Table 1). DCV derivative **7 a** showed an intensive absorption band with maximum at 561 nm which we address according to theoretical calculations (vide infra) to the HOMO‐LUMO transition with partial charge‐transfer (CT) character. The molar extinction coefficient was 120 700 M^−1^ cm^−1^ and the optical gap is calculated to 2.08 eV. The emission maximum appeared at 596 nm. The film absorption was red‐shifted and broadened exhibiting a maximum at 592 nm. Due to inductive effects, the introduction of hexyl side chains at the β‐positions of the SN5’ core resulted in a small bathochromic shift of 6 nm for **11 a** compared to **7 a**. However, when the acceptor strength of the end groups is increased from DCV in **11 a** to IND in **12 a** and to DCI in **13 a** a strong red‐shift from 567 to 625 and to 720 nm and hyperchromism concomitant with broadening of the main band is observed (Figure 4, left). In addition, the emission maxima were red‐shifted and the optical gap decreased from 1.85 to 1.50 eV. The corresponding film spectra showed the same trends with respect to the acceptor strength and were further red‐shifted and broadened compared to the corresponding solution spectra (Figure 4, right). As expected, replacement of the branched 2‐hexyldecyl side chain by *tert*‐butylbenzyl in **12 b** and **13 b** led to little deviation of the optical parameters compared to **12 a** and **13 a**, respectively. Comparison of the optical data of **7 a** to corresponding DCV‐substituted SN5 derivative (Figure 1) results in a blue‐shift of the solution absorption and emission maximum by about 20 nm each, whereas the film absorption is slightly red‐shifted by 4 nm.^[12]^

**Table 1:** Optical properties of acceptor‐substituted SN5’ derivatives. The absorption and emission spectra were measured in dichloromethane at room temperature, maxima are underlined, sh=shoulder.

SN5’	$\lambda_{max}^{sol}$ [nm]	*ϵ* [L mol^−1^ cm^−1^]	$E_{g}^{sol}$^[a]^ [eV]	$\lambda_{max}^{em}$ [nm]	$\lambda_{max}^{film}$^[b]^ [nm]	$E_{g}^{film}$^[a]^ [eV]
**7 a**	561, 529 (sh)	120 700	2.08	596	592, 546	1.96
**11 a**	567, 533 (sh)	125 660	2.06	600	592, 552	1.85
**12 a**	625, 577 (sh)	138 360	1.86	666	647 (sh), 607	1.74
**13 a**	720, 662 (sh)	168 000	1.61	768	755 (sh), 697	1.50
**12 b**	621, 582 (sh)	133 145	1.86	664	646 (sh), 593	1.72
**13 b**	715, 662 (sh)	174 360	1.61	764	757 (sh), 693	1.50
**16 c**	628, 567	n.d.^[c]^	1.80	685	642 (sh), 591 ^[d]^	1.74

[a] *E*_g_ calculated with 1240/*λ*_onset_. [b] Films were spin‐coated on glass slides from chloroform solution. [c] Not to determine because two electronic transitions are involved in the absorption band (vide infra). [d] The film was spin‐coated on glass slides from dichloromethane solution

**Scheme 4:**
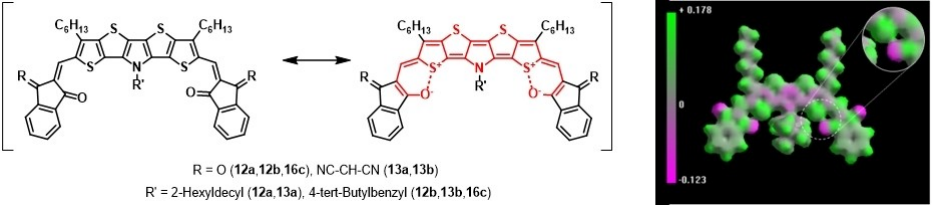
Left: Mesomeric structures of IND‐ and DCI‐substituted SN5’ derivatives **12 a**, **12 b**, **13 a**, **13 b**, and **16 c** representing stabilizing intramolecular, noncovalent S−O interactions. Right: Calculated electrostatic potential for **12 b** and, inset; a close‐up at the S−O coalescence point.

The redox properties of A‐D‐A SN5’ derivatives **7 a**, **11 a**, **12 a**–**b**, **13 a**–**b**, and dimeric **16 c** were studied by cyclic voltammetry (CV) and differential pulse voltammetry (DPV) in dichloromethane and tetrabutylammonium hexafluorophosphate (0.1 M) as electrolyte, potentials were referenced against the ferrocene/ferricenium couple (Fc/Fc^+^) and data is compiled in Table 2. For all derivatives in the series, we find a reversible oxidation wave in the potential regime of 0.48 to 0.87 V indicating the formation of stable radical cations delocalized on the heteropentacene core. Compared to **7 a**, introduction of hexyl side chains at the SN5’ core in **11 a** results in a slightly negative shift of the oxidative half‐wave potential (Δ*E*=0.06 V) due to their inductive electron‐donating effect of the alkyl groups. Unexpectedly, the oxidation potentials of the couples **12 a**–**13 a**, **12 b**–**13 b**, and dimer **16 c** are substantially negatively shifted with respect to DCV derivatives **7 a** and **11 a**, despite they bear the stronger electron‐withdrawing groups IND or DCI in the A‐D‐A system. We explain this issue with the analysis of mesomeric structures: one important contribution comes from an elongated conjugated π‐system due to intramolecular noncovalent S⋅⋅⋅O interactions of a carbonyl group of each IND‐ or DCI‐acceptor moiety with the sulfur of the terminal thiophene units in the SN5’ core (Scheme 4). Such S−O interactions have been found for other oligomeric^[30]^ or polymeric conjugated π‐systems and not only increase backbone planarity, but also might lead to enhanced photovoltaic performance.[^31^, ^32^] Nevertheless, the increased acceptor strength of DCI (**13 a/b**) versus IND (**12 a/b**) is reflected in increased oxidation potentials. In a second reversible one‐electron transfer step derivatives **12 a**–**13 a**, **12 b**–**13 b**, and **16 c** are oxidized at potentials around 1.2 V under the formation of heteropentacene dications.

**Table 2:** Summary of the redox properties of acceptor‐substituted SN5’ derivatives. Cyclic voltammograms in dichloromethane, tetrabutylammonium hexafluorophosphate (0.1 M), scan speed 100 mV/s, RT, potentials vs. ferrocene/ferricenium (Fc/Fc^+^) and calculated frontier orbital energy levels.

SN5’	*E*_1/2_^Ox1/Ox1’[a]^ [V]	E_1/2_^Ox2[a]^ [V]	E_1/2_^Red1[a]^ [V]	E_1/2_^Red2/3[a]^ [V]	HOMO [eV]^[b]^	LUMO [eV]^[b]^	*E*_g_ [eV]^[c]^
**7 a**	0.89		−1.21	−1.61	−5.89	−3.99	1.90
**11 a**	0.83		−1.42	−1.85	−5.78	−3.81	1.97
**12 a**	0.48 (0.64)	1.18	−1.33	−1.55	−5.48	−3.87	1.61
**13 a**	0.64	1.20	−0.99	−1.20/−1.65	−5.61	−4.20	1.41
**12 b**	0.53 (0.74)	1.18	−1.25	−1.46	−5.55	−3.89	1.66
**13 b**	0.70	1.24	−0.91	−1.17	−5.67	−4.17	1.50
**16 c**	0.56 (0.77)	1.19	−1.28	−1.44	−5.56	−3.85	1.71

[a] Half‐wave potentials *E*_1/2_ of redox waves were determined via differential pulse voltammetry (DPV). [b] Calculated from the onset values of the first oxidation and reduction wave; Fc/Fc^+^ was set to −5.1 eV vs. vacuum (*E*_HOMO_=−5.1 eV−*E*_on,Ox_; *E*_LUMO_=−5.1 eV−*E*_on,Red_). [c] *E*_g_=*E*_LUMO_−*E*_HOMO_.

In the reductive potential regime, the SN5’ derivatives typically showed two quasi or irreversible reduction waves, which we address to the sequential formation of radical anions on the acceptor subunits in the A‐D‐A system. The reduction potentials in the series **11 a** (−1.36 V), **12 a** (−1.32 V), and **13 a** (−1.04 V) correlate with the strength and electron‐withdrawing character of the terminal acceptor groups (DCV>IND>DCI) and continuously are shifted to more positive potentials. The electrochemical energy gaps consequently follow the same trend and become smaller with increasing acceptor strength which is in accordance with the optical data.

**Figure 5:**
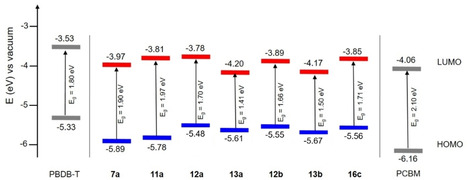
Energy‐level diagram showing the HOMO (blue) and LUMO (red) energy levels and energy gaps *E*_g_ of acceptor‐substituted SN5’ derivatives in comparison to donor polymer PBDB‐T and acceptor PCBM.

In order to get information about the energetics of the frontier orbitals, we determined HOMO and LUMO energy levels, which are important for application of the molecules in organic solar cells, from the onset of the first oxidation and first reduction wave, respectively. A schematic energy level diagram of the frontier orbitals and the corresponding band gaps *E*_g_ was derived and is shown in Figure 5. The HOMO and LUMO energies in the series of investigated SN5’ derivatives are compared to D‐A polymer PBDB‐T and fullerenes PCBM which were used in the photovoltaic experiments (vide infra) and correlate to the trends seen in the redox behavior.

Nevertheless, it becomes clear that due to their LUMO energies derivatives **7 a**, **11 a**, **12 a**, **12 b**, and **16 c** should allow for their use as donor component in BHJ‐OSCs when fullerenes are applied as acceptor and should guarantee photoinduced electron transfer from the donor to the acceptor. In contrast, the LUMO energies of DCI derivatives **13 a** and **13 b** are too low for a donor component, but on the other hand all SN5’ derivatives should be applicable as NFA in combination with donor polymer PBDB‐T, because their lower LUMO energies should provide sufficient energy offset for electron transfer.

### Deeper insights into the optoelectronic properties of SN5’ derivative 12 b in comparison to corresponding dimeric 16 c supported by quantum chemical calculations

**Figure 6:**
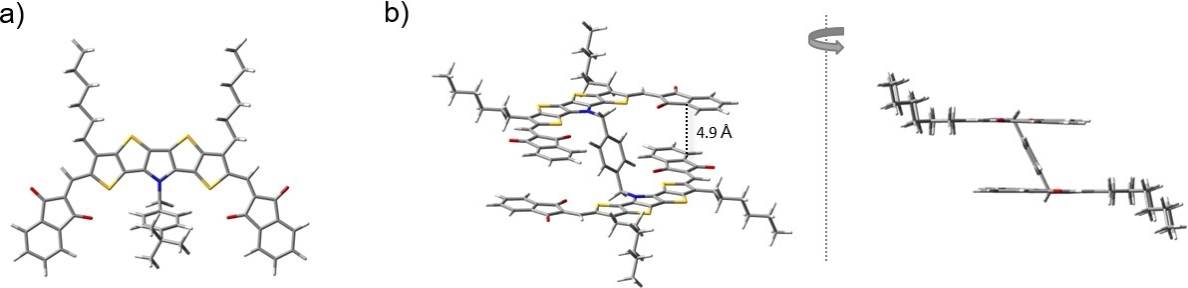
Calculated optimized geometries of derivatives a) **12 b** and b) **16 c** (rotated and side view).

The effect of connecting two SN5’ units through a xylylene spacer in dimer **16 c** on the electronic properties in comparison to “monomeric” SN5’ **12 b** is rather subtle and can be explained with the help of quantum chemical calculations (semiempirical PM6, DFT (CAMB3LYP), and TD‐DFT). IND‐substituted **12 b** shows a coplanar geometry of the A‐D‐A conjugated π‐system including the alkyl chains (Figure 6a). The 4‐*tert‐*butylbenzyl substituent at the central nitrogen atom protrudes out of the molecular plane. The acceptor units are oriented in a *syn* conformation with respect to the terminal thiophenes leading to a crescent shaped conformation as it was also observed in the case of comparable DCV derivatives.[^13^, ^20^] The distance between the terminal sulfur atoms in the SN5’ unit and the closest oxygen atoms from IND is with 2.48 Å far below the sum of the van der Waals radii (3.32 Å) indicating a nonbonding interaction between the two atoms. This finding is corroborated by the mapped isosurface of the electrostatic potential (Figure 4, right) and supports the mesomeric structures postulated in Figure 4, left.

**Figure 7:**
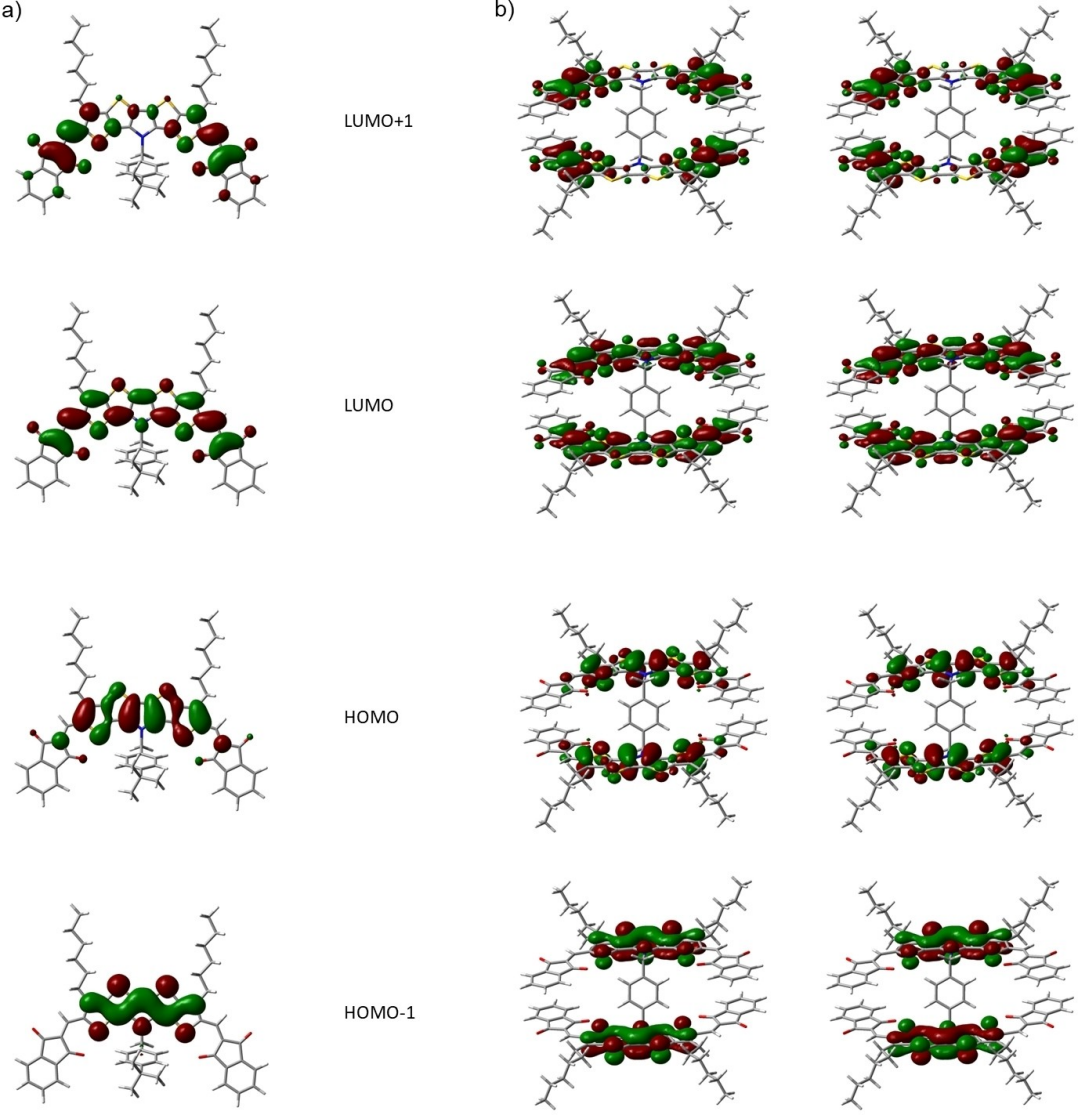
Calculated electron‐density maps of the frontier orbitals for a) **12 b** and b) **16 c** (degenerated levels).

The calculated lowest energy geometry of dimer **16 c** shows an antiparallel arrangement of the two planar conjugated SN5’ π‐systems, whereby the acceptor units arrange stack‐like face to face at distances of about 4.9 Å (Figure 6b). The alkyl chains bend out in opposite direction of the corresponding SN5’ subunit. The short nonbonding S‐O atom distances are also present in the optimized geometry of the lowest energy conformation of **16 c**. Although a careful analysis on the hyperpotential surface was performed, no other conformations, for example as a result of the rotation of the two subunits with respect to each other, could be stabilized. The electronic density maps of the frontier orbitals of both derivatives are shown in Figure 7. In case of dimer **16 c** the orbitals are degenerated due to the symmetry of the molecule. In both derivatives, the HOMO and HOMO‐1 is mostly localized on the SN5’ core unit whereas the unoccupied molecular orbitals spread out to the acceptor units (LUMO) or are mostly localized on them (LUMO+1).

**Figure 8:**
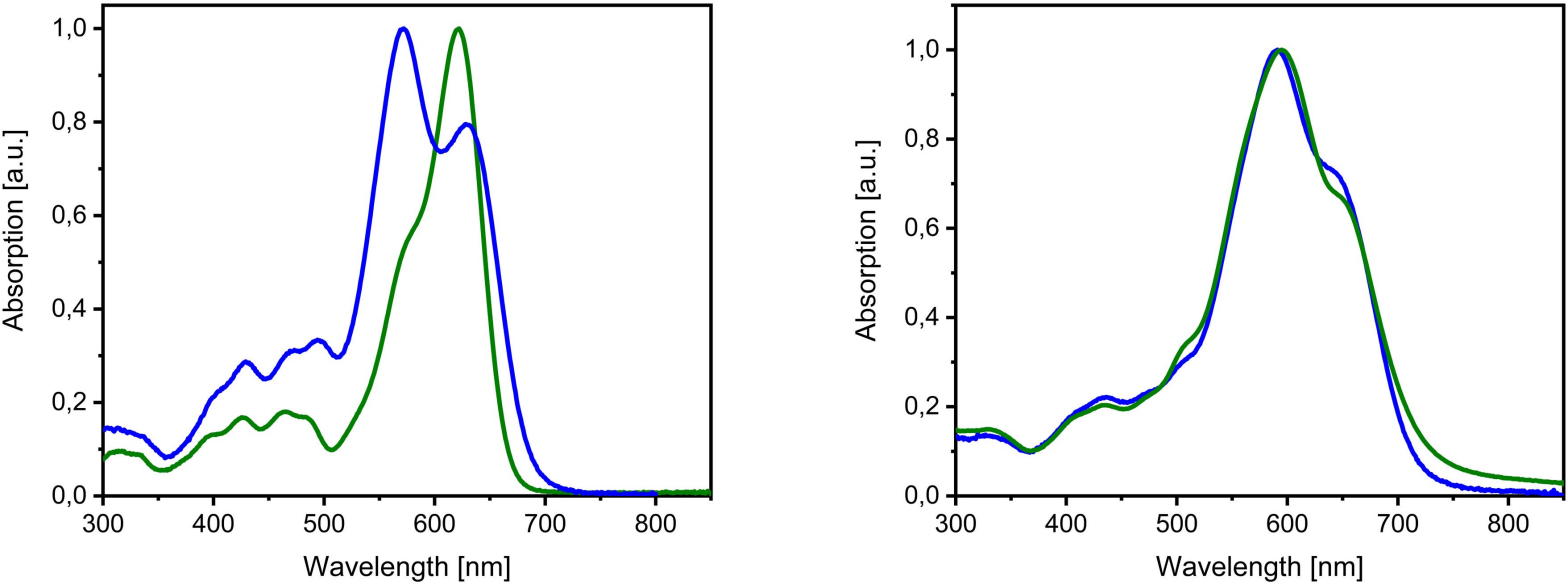
Normalized absorption spectra of the SN5’ derivative **12 b** (green) and SN5’ dimer **16 c** (blue) recorded in CH_2_Cl_2_ (left) and in thin films doctor‐bladed from chloroform solution (right).

The low‐energy optical transitions were computed on the converged molecular geometries with TDDFT methods. The three first transitions in the case of **12 b** appear at 600, 415, and 370 nm. The band at lowest energy corresponds to a HOMO→LUMO transition with strong oscillator strength and transition dipole moment parallel to the molecular axis. It well correlates with the experimentally observed band, whose absorption maximum is localized at 621 nm (Figure 8, left, green curve). The calculated probability of the second (HOMO→LUMO+1) and third transition (HOMO→LUMO+2) at 415 and 370 nm is much lower and in accordance with the smaller absorption bands in the 350–500 nm energy range of the depicted absorption spectrum.

In the case of dimer **16 c**, the calculated transitions appear at 625, 557, and 404 nm. In contrast to **12 b**, the band at lowest energy features substantially lower oscillator strength and can be characterized as a combination of HOMO→LUMO and HOMO‐1→LUMO+1 transitions. A second electronic transition at 557 nm exhibits a very strong oscillator strength and comprises a combination of the HOMO→LUMO+1 and the HOMO‐1→LUMO transitions. A very good correlation with the experimentally observed absorption peaks at 628 nm (less intense) and 567 nm (stronger) is obtained (Figure 8, left, blue curve) well explaining the broadness of the absorption band of **16 c** by the sum of two electronic transitions in this energy region in contrast to the monotonic transition in the case of **12 b**.

The absorption spectra in solution of both derivatives **12 b** and **16 c** evidence concentration dependent aggregation. Starting at a concentration of 10^−5^ M, the absorption spectrum of **12 b** showed a growing band at ∼570 nm (Figure S15, top). In the case of derivative **16 c**, the changes in the absorption spectrum start at lower concentrations (∼10^−6^ M) most probably due to the preformed, less flexible geometry of the subunits in the dimer. The peak at low energy evolves with increasing concentration to a shoulder which indicates loss of molecularly dissolved material. Simultaneously an increasing absorption can be seen at the same wavelength (∼570 nm) than the one observed for **12 b** (Figure S15, bottom). Because both derivatives are composed of the same chromophore, we can attribute the hypsochromically shifted band appearing at higher concentrations to the formation of H‐type aggregates of antiparallelly aligned π‐conjugated SN5’ cores. Accordingly, the normalized thin film absorption spectra of both derivatives became nearly identical and we conclude that the chromophores in films of monomer **12 b** and dimer **16 c** are arranged in the same antiparallel fashion (Figure 8, right).

**Figure 9:**
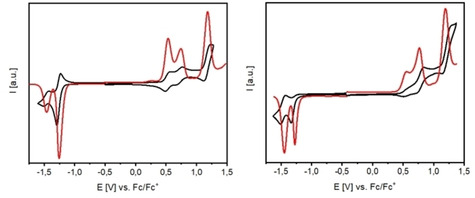
Cyclic voltammograms (black) and overlayed differential pulse voltammograms (red) of IND‐SN5’ derivatives **12 b** (left) and dimer **16 c** (right) measured in dichloromethane, tetrabutylammonium hexafluorophosphate (0.1 M), scan speed 100 mV/s, RT, potentials referenced against the redox couple ferrocene/ferricenium (Fc/Fc^+^) as internal standard.

The oxidation behavior of “monomeric” derivative **12 b** and dimeric **16 c** revealed more complex CVs and DPVs than the rest of the molecules investigated in the series and showed visibly three oxidation waves. In contrast, those of corresponding DCI derivatives **13 b** are less resolved and showed the expected two waves. We attribute the first wave or couple of waves around 0.5–0.8 V to the formation of radical cations and the stronger wave at ∼1.2 V to the formation of dications. In Figure 9 the cyclic voltammograms and overlaid DPVs of **12 b** in comparison to **16 c** are depicted.

**Figure 10:**
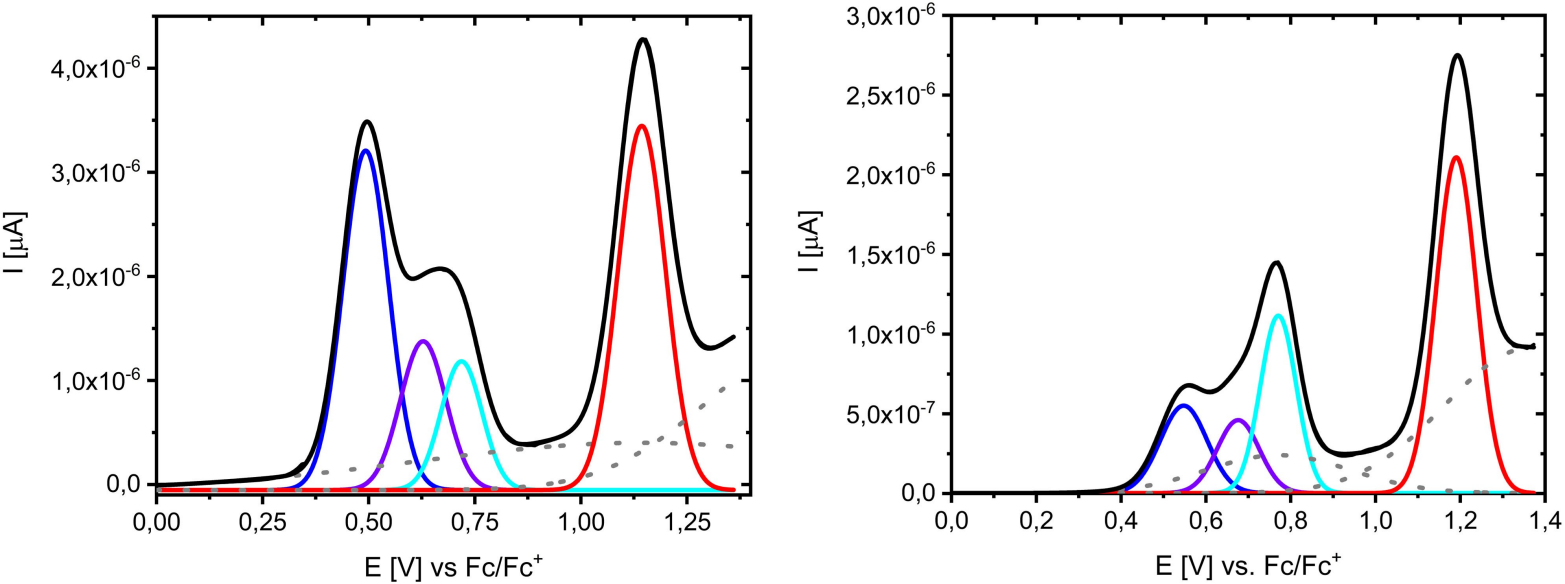
Deconvolution of the DPV curve (black) of IND derivative **12 b** (left) and of dimer **16 c** (right).

We analyzed this phenomenon for **12 b** and dimeric **16 c** by deconvolution of the DPV curves in the potential regime of 0 to 1.4 V. For monomer **12 b** the best fitting (*R* ^2^=0.99976) was obtained when in total four bands with peak potentials at *E*_1_=0.49 V (blue curve), *E*_1_′=0.63 V (violet), *E*_1_′′=0.72 V (cyan), and *E*_2_=1.14 V (red) were evolved (Figure 10, left). Attempts to fit the deconvolution by means of only three bands failed. The relative integrated areas, normalized to the larger fourth peak, result in a ratio of approximately 7 : 3 : 2.5 : 10. Deconvolution of the DPV curve of dimer **16 c** with two electrochemically active units led to similar results, whereby the potentials of the four peaks (*E*_1_=0.55 V, *E*_1_′=0.68 V, *E*_1_′′=0.77 V, *E*_2_=1.19 V) are slightly positively shifted and the relative intensities vary compared to **12 b**. The integrated areas were determined to a ratio of approximately 3 : 2 : 5 : 10 (Figure 10, right).

**Scheme 5:**
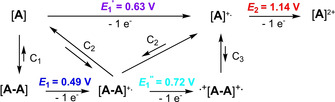
Schematic presentation of the stepwise oxidation (*E*_1_‐*E*_2_) including chemical equilibria (C_1_‐C_3_) of IND derivatives **12 a**, **12 b**, and **16 c** (generally represented as A, values are taken for **12 b**).

We address this feature to the superimposition of electrochemical processes (E) with chemical (C) association/dissociation equilibria of monomeric to dimeric species and discuss it generalized for **12 b** and **16 c**, which are represented by “A” (Scheme 5). According to the findings in absorption spectroscopy, we assume that in the chemical equilibrium C_1_ neutral [A] partly aggregates to neutral dimer [A‐A], which is first oxidized (*E*_1_ blue band) to the monocharged dimer [A‐A]^.+^ whereas [A] is oxidized to [A]^+.^ at slightly higher potential (*E*_1_′ violet band). The dimeric radical cation [A‐A]^.+^ is interconnected with a second association equilibrium (C_2_) and reversible dissociation into neutral [A] and monomeric radical cation [A]^.+^ due to Coulombic repulsion. Further oxidation of [A‐A]^.+^ at more positive potential leads to doubly oxidized radical dication ^+.^[A‐A]^.+^ (*E*_1_′′ cyan band), which in the third chemical equilibrium C_3_ dissociates in two equivalents of monomeric radical cation [A]^.+^. It is long known for oligothiophene radical cations that they dimerize in temperature‐dependent equilibria whereby the dimers are favored at lower temperatures.[^33^, ^34^] The fourth oxidation step at distinctly higher potential (*E*_2_ red band) is correlated with the formation of stable monomeric dications [A]^2+^. The phenomenon of electron transfer coupled to chemical association was previously already identified for thiophene‐based macrocycles^[35]^ and catenanes.^[36]^

We furthermore studied the redox behavior of **12 b** and **16 c** depending on concentration and temperature. The analysis of intensities and areas of the DPV bands are summarized in Figure S16 and corroborates the above formulated complex reaction scheme.

### Photovoltaic properties and solar cell performance of SN5’ derivatives

DCV‐substituted SN5 derivatives reached PCEs of up to 6.5 % in vacuum‐processed bulk‐heterojunction organic solar cells (BHJ‐OSC) when used as photoactive donor in blends with C_60_ as acceptor.^[12]^ The series of A‐D‐A SN5’ derivatives now provides strong absorbers, which due to their variable LUMO and HOMO energy levels (Figure 5) should be applicable either as donor in blends with soluble fullerene PC_61_BM or PC_71_BM as acceptor or as nonfullerenic acceptor (NFA) with donor polymer PBDB‐T.^[37]^ In this respect, SN5’ derivatives **7 a**, **11 a**, **12 a**, **12 b**, and **16 c** firstly were implemented and optimized in solution‐processed BHJ‐OSCs with the standard device structure glass/ITO/PEDOT : PSS/**SN5’** : PCBM/LiF/Al. The solar cells were prepared under ambient conditions by spin‐coating solutions of the respective D‐A blends in various solvents and were individually optimized. The processing parameters were varied with respect to processing solvents, layer thickness, D : A ratio, and additives or post‐treatment by solvent vapor annealing (SVA). In Table 3, a summary of the best devices of the SN5’ heteropentacenes used as donor and PC_61_BM or PC_71_BM as acceptor is presented. Their use as donor is justified by their LUMO energy levels (*E*_LUMO_=−3.78 to −3.97 eV), which in each case show a sufficiently large energy offset with respect to the LUMO of the PCBM acceptors (*E*_LUMO_≈−4.06 to −4.1 eV)^[38]^ guaranteeing electron transfer from the excitonic state of the D to the LUMO of the A at the D‐A interface. However, the LUMO levels of DCI derivatives **13 a** and **13 b** were below this of PCBM opposing their application as donor in BHJ‐OSCs with PCBM as acceptor, because no driving force for charge transfer from donor to acceptor would be given.

**Table 3:** Photovoltaic parameters of A‐D‐A SN5’ derivatives **7 a**, **11 a**, **12 a**, **12 b**, and **16 c** as donor with PC_61_BM or PC_71_BM as acceptor.

SN5’ donor	Acceptor	D : A ratio	SVA/ additive	*V*_OC_ [V]	*J*_SC_ [mA/cm^2^]	FF	PCE [%]
**7 a** ^[a]^	PC_61_BM	1 : 2	−	0.74	0.43	0.34	0.11
**11 a** ^[a]^	PC_61_BM	1 : 2	−	0.96	0.24	0.28	0.06
**12 a** ^[b]^	PC_61_BM	1 : 1.25	−	1.11	8.68	0.44	4.25
**12 a** ^[b]^	PC_61_BM	1 : 1.25	THF 15 s	1.06	9.67	0.56	**5.49**
**12 a** ^[b]^	PC_71_BM	1 : 1.5	THF 25 s	1.04	5.18	0.44	2.37
**12 b** ^[b]^	PC_61_BM	1 : 2	−	1.08	9.18	0.43	4.25
**16 c** ^[c]^	PC_61_BM	1 : 1	THF 30 s	0.92	2.72	0.40	0.93

Best values after optimization, device architecture: glass/ITO/PEDOT:PSS/SN5’‐PCBM/LiF/Al; processing conditions: spin‐coating [a] @2000 rpm, chloroform solution of 15 mg/mL at RT; [b] @1000 rpm, chloroform solution of 15 mg/mL at RT; [c] @ 1500 rpm, tetrachoroethane solution of 15 mg/mL at 80 °C.

As a result, optimized devices with DCV‐terminated SN5’ derivatives **7 a** and **11 a** as donor and acceptor PC_61_BM in a ratio 1 : 2 gave poor efficiencies far below 1 % due to low photocurrents (*J*_SC_) and fill factors (FF). IND‐derivatized SN5’ **12 a** blended with PC_61_BM in a ratio of 1 : 1.25 achieved much better results with strongly increased photovoltaic parameters. A very high open circuit voltage (*V*_OC_) of 1.11 V contributed to a PCE of 4.25 %, which could be improved to 5.49 % by post‐treatment of the photoactive layer by SVA in THF (Figure S17). The application of PC_71_BM as acceptor surprisingly reduced the performance to 2.37 %. The exchange of the 2‐hexyldecyl side chain in **12 a** to *p*‐*tert*‐butylbenzyl in **12 b**, which was blended with PC_61_BM in a ratio 1 : 2, gave nearly the same results before SVA (PCE 4.25 %). In this case, SVA however did not further improve the performance. Finally, dimeric SN5’ derivative **16 c**, which comprised the same electronically active units than **12 b**, underperformed and gave a PCE of less than 1 %. Hence, this investigation revealed that IND‐substituted derivative **12 a** is far better suited as donor in biphasic BHJ‐OSCs than the DCV derivatives and showed the best performance in the series, which is comparable to a similar indandione‐substituted septithiophene,^[39]^ but could not reach the best oligomer/PCBM solar cells.^[40]^

External quantum efficiency (EQE) spectra give information on the charge generation process and reached more than 70 % in the regime of 550–600 nm for BHJ‐OSCs of **12 a**/PC_61_BM showing that most of the contribution comes from donor IND‐SN5’ **12 a** (Figure S19).

Due to the lower lying LUMO energies (*E*_LUMO_=−3.78 to −4.20 eV), we tested and optimized SN5’ derivatives **7 a**, **11 a**, **12 a**, **13 a**, and **13 b** as NFA in blends with donor polymer PBDB‐T (*E*_LUMO_=−3.53 eV) in the same device structure: glass/ITO/PEDOT : PSS/PBDB‐T : **SN5’**/LiF/Al (Table 4). Devices with SN5’ **7 a** and polymer PBDB‐T in a ratio 3 : 2 and with 1,8‐diiodooctane (DIO) as additive gave a PCE of 2.7 %. The structurally related **11 a** showed an increased open‐circuit voltage (*V*_OC_) of 1.14 V, but a strongly decreased photocurrent (*J*_SC_) and fill factor (FF) leading to a low PCE of only 0.28 %. Here, the presence of hexyl side chains at the SN5’ core obviously leads to a quite different and less favorable morphology in the photoactive blend layer. With increasing electron‐withdrawing strength of the terminal acceptors in **12 a** and **13 a** and concomitant lowering of the LUMO energy levels, expectedly *V*_OC_, which theoretically corresponds to the energy difference of the HOMO of the donor and the LUMO of the acceptor, decreases from 1.14 V (**11 a**) to 1.16 V (**12 a**) and to 0.87 V (**13 a**). In contrast, *J*_SC_ and simultaneously PCE are gradually increased to reach the best PCE of 3.51 % for **13 a** as NFA (Figure S18). For BHJ‐OSCs of **13 a**/PBDB‐T, the EQE with around 30 % is significantly lower compared to **12 a**/PC_61_BM but rather uniformly extended from 400 to over 800 nm showing equal contributions of donor and SN5’ NFA (Figure S18).

**Table 4:** Photovoltaic parameters of A‐D‐A SN5’ derivatives as nonfullerenic acceptor with donor polymer PBDB‐T.

SN5’ NFA	Donor	D : A ratio	Additive	*V*_OC_ [V]	*J*_SC_ [mA/cm^2^]	FF	PCE [%]
**7 a** ^[a]^	PBDB‐T	2 : 3	DIO 0.5 %	0.90	5.14	0.58	2.70
**11 a** ^[b]^	PBDB‐T	2 : 3	DIO 1 %	1.14	0.79	0.32	0.28
**12 a** ^[c]^	PBDB‐T	1 : 2	DIO 0.75 %	1.06	3.04	0.37	1.18
**13 a** ^[d]^	PBDB‐T	2 : 3	DIO 0.25 %	0.87	9.03	0.45	**3.51**
**13 b** ^[e]^	PBDB‐T	1 : 1	DIO 0.25 %	0.71	0.60	0.42	0.16

Best value after optimization; device architecture: glass/ITO/PEDOT:PSS/PBDB‐T:SN5’/LiF/Al; processing conditions: spin‐coating [a] @2000 rpm, chloroform solution of 10 mg/mL at 50 °C; [b] @3000 rpm, chlorobenzene solution of 15 mg/mL at 80 °C; [c] @3000 rpm, chlorobenzene solution of 20 mg/mL at 80 °C; [d] @3000 rpm, tetrachloroethane solution of 20 mg/mL at 80 °C; [e] @3000 rpm, tetrachloroethane solution of 15 mg/mL at 80 °C.

By the successful application of A‐D‐A SN5’ derivatives as both, donor and NFA, their versatility as electronic material in BHJ‐OSCs has been demonstrated. In particular, IND‐substituted SN5’ **12 a** has shown the most flexible applicability with a PCE of 5.49 % as donor and of 1.18 % as NFA, respectively.

## Conclusion

In summary, we have presented the synthesis and characterization of novel functionalized *S,N*‐heteropentacenes SN5’ and their application in organic solar cells. The structural variation included the substituent at the inner pyrrole nitrogen and the acceptor units in the A‐D‐A‐type conjugated π‐system. The series was characterized with respect to their optical and redox properties, and valuable structure‐property relationships were obtained. The thereby tunable frontier orbital energies allowed a rare twofold application of the functionalized *S,N*‐heteropentacenes: they were either implemented as donor in solution‐processed BHJ‐OSCs or as nonfullerenic acceptor reaching respectable PCEs of 5.49 % and 3.51 %, respectively.

## Conflict of interest

The authors declare no conflict of interest.

## Supporting information

As a service to our authors and readers, this journal provides supporting information supplied by the authors. Such materials are peer reviewed and may be re‐organized for online delivery, but are not copy‐edited or typeset. Technical support issues arising from supporting information (other than missing files) should be addressed to the authors.

Supporting Information
