# Supplementary material for: Advanced Acceptor‐Substituted S,N‐Heteropentacenes for Application in Organic Solar Cells
Source: Chemistry. 2021 May 29;27(42):10913–24. doi: 10.1002/chem.202100702 (PMC8362193; doi:10.1002/chem.202100702)
Supplement: Supplementary file 1 — Supporting Information [file CHEM-27-10913-s001.pdf]

Chemistry–A European Journal

Supporting Information

Advanced Acceptor-Substituted *S*,*N*-Heteropentacenes for Application in Organic Solar Cells

Teresa Kraus, Sebastian Lucas, Pascal Wolff, Anna Aubele, Elena Mena-Osteritz, and Peter Bäuerle*

Table of Content

Experimental Section	page
1. Instruments and measurements	S2
2. Materials	S3
3. Synthesis	S4
4. ^{1}H-, ^{13}C-NMR spectra	S14
5. High resolution mass spectra	S20
6. Additional absorption spectra and DPV	S23
7. Organic photovoltaic data	S24
8. References	S25

Experimental Section

1. Instruments and measurements. NMR spectra were recorded on a Bruker AMX 500 (^{13}C-NMR: 125 MHz; ^{1}H-NMR: 500 MHz) or an Avance 400 Spectrometer (^{13}C-NMR: 100 MHz; ^{1}H-NMR: 400 MHz). Chemical shift values (δ) are expressed in parts per million (ppm) using residual solvent protons (δ= 3.58 (THF-d_8), 5.32 (CD_2Cl_2), 2.50 (DMSO-d_6) and 7.26 ($CDCl_3$) for ^{1}H-NMR spectra and δ= 67.21 (THF-d_8), 53.84 (CD_2Cl_2), 39.52 (DMSO-d_6) and 77.16 ($CDCl_3$) for ^{13}C-NMR spectra) as internal standard. Coupling constants *J* relate to proton-proton couplings. The splitting patterns are designated as follows: s (singlet), d (doublet), t (triplet), and m (multiplet). The assignments of the protons correspond to TT-α-*H* (α-proton of thienothiophene unit), TT-β-*H* (β-proton of thienothiophene unit), Th-α-*H* (α-proton of terminal thiophene unit), Th-β-*H* (β-proton of terminal thiophene unit), o-/m-Ph-*H* (ortho-/meta-phenyl proton). Thin layer chromatography was carried out on aluminum plates, pre-coated with silica gel, Merck Si60 F_{254}. Preparative column chromatography was performed on glass columns packed with silica gel 60 M (Macherey-Nagel) particle size 0.04-0.063 mm or silica gel 60 (Macherey-Nagel) particle size 0.063-0.2 mm. Melting points were determined using a Mettler Toledo DSC 823e under Ar flow (heating rate 10 °C/min) or a Büchi Melting Point M-565 (not corrected). GC-MS measurements were performed on a Shimadzu GCMS-QP2010 SE. MALDI-TOF mass spectra were measured on Bruker Daltonik Reflex III and high resolution MALDI mass spectra were performed on a Bruker SolariX using trans-2-[3-(4-*tert*-butylphenyl)-2-methyl-2-propenylidene]malononitrile (DCTB) as matrix. CI mass spectra were recorded on a Finnigan MAT, SSQ-7000. EI mass spectra were recorded on a Varian Saturn 2000 GC-MS. High resolution APCI spectra were performed on a Bruker SolariX using acetonitrile as solvent.

Optical measurements in solution were carried out in 1 cm cuvettes with Merck Uvasol grade solvents. Absorption spectra were recorded on a Perkin Elmer Lambda 19 spectrometer and corrected fluorescence spectra were recorded on a Perkin Elmer LS 55 fluorescence spectrometer. Cyclic voltammetry experiments were performed with a computer-controlled Autolab PGSTAT30 potentiostat in a three-electrode single-compartment cell (3 mL). The platinum working electrode consisted of a platinum wire sealed in a soft glass tube with a surface of A = 0.785 mm^2, which was polished down to 0.25 µm with Buehler polishing paste prior to use to guarantee reproducible surfaces. The counter electrode consisted of a platinum wire and the reference electrode was an Ag/AgCl reference electrode. All potentials were internally referenced to the ferrocene/ferricenium couple (Fc/Fc^+). For the measurements, concentrations of 10^{-3} M of the electroactive species were used in freshly distilled and deaerated dichloromethane (Lichrosolv, Merck) purified with a Braun MB-SPS-800 and 0.1 M $(n\text{-}Bu)_4NPF_6$ (Fluka; recrystallized twice from ethanol).

Device fabrication of organic solar cells was performed with indium tin oxide (ITO) patterned glass substrates (15 Ω cm^{-2}, from Naranjo Substrates), which were precleaned with acetone, Mucasol soap solution, and isopropanol, before treated in a UV-ozone cleaner for 30 min. PEDOT:PSS was spin-coated at 3000 rpm to obtain thin films of 20-30 nm thickness. The active layer solutions (10 mg/mL to 20 mg/mL in chloroform, chlorobenzene or tetrachloroethane) were deposited by spin-coating at various spin speeds. Thin films of lithium fluoride (LiF, 0.7 nm) and aluminum (Al, 100-120 nm) were deposited by high-vacuum evaporation at pressures below 3 x 10^{-6} mbar (Nano 36, from Kurt J. Lesker Co.). Solvent vapor annealing (SVA) was performed by a literature known procedure.[1] One substrate contained four photoactive areas of 0.09 and 0.16 cm^2. *J-V*-characteristics of the prepared devices were obtained by using a solar simulator (Oriel Instruments, class AAA, AM 1.5G, 10 $mWcm^{-2}$). Apart from LiF and aluminum deposition, processing and characterization of the solar cells were performed under ambient conditions. The external quantum efficiency (EQE) was measured under monochromatic light from a 300 W Xenon lamp in combination with a monochromator (Oriel, Cornerstone 260), modulated with a mechanical chopper. The response was recorded as the voltage over 220 Ω resistance using a lock-in amplifier (Merlin 70104). A calibrated Si-cell was used as a reference (Newport 70356-70316NS). Active layer thickness was determined by profilometer Veeco Dektak 150 High Performance Surface Profiler.

2. Materials. Toluene (Sigma Aldrich), THF (Carl Roth GmbH), dichloromethane, DMF (VWR), and diethylether (VWR) were dried and purified by a MB SPS-800 (MBraun). Dichloromethane, chloroform, dimethylformamide, petroleum ether, methanol, and acetone were purchased from VWR, chlorobenzene from Merck and distilled prior to use. Heptanoylchloride, cesium carbonate, bis(dibenzylideneacetone)palladium(0), triisopropylsilylchloride, sodium *tert*-butanolate, 4-*tert*-butylbenzylamine and tetrabutylammonium fluoride were purchased from Sigma Aldrich. Aluminum trichloride, 1,3-indandione, piperidine, ferrocene, 1,2-dichloroethane, and 1,1,2,2-tetrachloroethane were bought from Alfa Aesar. Ethylthioglycolate, copper powder, phosphoryl chloride, sodium hydroxide, ammonium acetate, chlorobenzene, sodium hydroxide, potassium hydroxide, lithium hydroxide, sodium *tert*-butoxide, triisopropylsilyl chloride, diisopropylamine, quinoline and copper powder were purchased from Merck. *n*-BuLi (1.6 M in hexane) was purchased from Acros Organics and 1,1'-bis(diphenylphosphino)ferrocene from Fluorochem. Calcium hydride and 1,4-phenylendimethanamine were bought from TCI and magnesium sulfate from Grüssing. Malonodinitrile was purchased from Sigma Aldrich. Poly(3,4-ethylendioxythiophene) polystyrolsulfonate (PEDOT-PSS, Clevios P, VP.AI 4038 aqueous solution) was bought at Heraeus, [6,6]-Phenyl-C_{61}-butyric acid methyl ester and [6,6]-phenyl-C_{71}-butyric acid methyl ester were purchased at SolenneBV. Poly[(2, 6-(4,8-bis(5-(2-ethylhexyl)thien-2-yl)-benzo[1,2-*b*:4,5-*b′*]dithiophene))-alt-(5,5-(1′,3′-di-2-thienyl-5′,7′-bis(2-ethylhexyl)benzo[1′,2′-*c*:4′,5′-*c′*]dithiophene-4,8-dione)] (PBDB-T) was purchased from Ossila, 1,8-diiodooctane (DIO) from Alfa Aesar, ultrapure aluminum (99.98%) from Umicore, and lithium fluoride from ACROS. 2-Brom-4-[tri(isopropyl)silyl]thieno[3,2-*b*]thiophene **1** and (3,3'-dibromo-[2,2'-bithieno[3,2-*b*]thiophene]-5,5'-diyl)bis(triisopropylsilane) **2**,[2,3] 2-hexyldecylamine **3a**,[4] 3,3'-dibromo-6,6'-dihexyl-2,2'-bithieno[3,2-*b*]thiophene **8**,[5] and dicyanomethylene-3-indanone (DCI)[6] were internally synthesized according to literature-known procedures.

3. Synthesis.

[3,3'-Dibromo-(2,2'-bithieno[3,2-*b*]thiophene)-5,5'-diyl]bis(triisopropylsilane) 2: For the preparation of the LDA-solution, freshly distilled diisopropylamine (0.29 mL, 2.1 mmol) was dissolved in THF (3mL) was cooled to 0 °C and *n*-butyl lithium (1.20 mL, 1.91 mmol, 1.6 M in *n*-hexane) was slowly added. The solution was stirred for another hour at 0 °C under argon and (6-bromothieno[3,2-*b*]thien-2-yl)triisopropylsilane **1** (670 mg, 1.78 mmol) in dry THF (12 mL) was subsequently added. Afterwards, the mixture was cooled to -78 °C, stirred for 30 min

and dry $CuCl_2$ ((257 mg, 1.91 mmol) was added to the mixture. The reaction thawed to room temperature overnight. Water was poured into the mixture and it was extracted with diethyl ether (3x50 mL). The mixture was purified over column chromatography (Flash, PE) which afforded (3,3'-dibromo-[2,2'-bithieno[3,2-*b*]thiene]-5,5'-diyl)bis(triisopropylsilane) (567 mg, 0.757 mmol, 85%) as a colorless solid. The product was further purified via recrystallization from methanol/PE. M.p. 226.3 °C (DSC). ^{1}H-NMR (400 MHz, [D_8]THF, 20 °C): δ=7.63 (s, 2H, TT-β-*H*), 1.38-1.51 (m, 6H, Si-(C*H*)$_3$), 1.18 (d, ^3J=7.5 Hz, 36H, Si-(CH)$_3$-(C*H*$_3$)$_6$) ppm. ^{13}C-NMR (100 MHz, [D_8]THF, 20 °C): δ=146.9, 141.4, 140.3, 132.4, 129.5, 105.5, 19.1, 12.9 ppm. HRMS (FTICR-MALDI): *m/z* calcd. for $C_{30}H_{44}Br_2S_4Si_2$ [M]$^+$ 748.02062; found 748.01989 (δm/m=0.98 ppm). The analytical data is in accordance with the literature.[2,3]

9-(2-Hexyldecyl)-2,7-bis(triisopropylsilyl)-9H-thieno[2',3':4,5]thieno[3,2-*b*]thieno[2',3':4,5]-thieno[2,3-*d*]pyrrole (4a): Under argon in a dry Schlenk tube, (3,3'-dibromo-[2,2'-bithieno[3,2-*b*]thien]-5,5'-diyl)bis(triisopropylsilane) (580 mg, 0.77 mmol), Pd(dba)$_2$ (45 mg, 0.08 mmol), dppf (172 mg, 0.31 mmol), and NaO^{t}Bu (744 mg, 7.74 mmol) were dissolved in dry toluene (41 mL). This solution was degassed with argon for 30 min and then treated dropwise with 2-hexyldecan-1-amine **3a** (280 mg, 1.16 mmol). After stirring the reaction mixture at 110 °C overnight, water was added, and the mixture was extracted with PE (2x 50 mL) and DCM (2x 50 mL). The combined organic layers were dried over Na_2SO_4 and the solvents were removed under reduced pressure. Column chromatography (silica gel, PE) of the crude product provided TIPS-protected SN5’ **4a** (530 mg, 0.64 mmol, 83%) as a yellowish waxy solid. ^{1}H-NMR (400 MHz, [D_8]THF, 20 °C): δ=7.53 (s, 2H, TT-β-*H*), 4.35 (d, ^3J=7.6 Hz, 2H, N-C*H*$_2$), 2.19-2.31 (m, 1H, N-CH$_2$-C*H*), 1.16-1.52 (m, 66H; C*H*$_2$, Si-(C*H*)$_3$, Si-(CH)$_3$-(C*H*$_3$)$_6$), 0.78-0.92 (m, 6H, CH$_2$-C*H*$_3$, CH$_2$-C*H*$_3$) ppm. ^{13}C-NMR (100 MHz, [D_8] THF, 20 °C): δ=142.0, 136.0, 134.0, 130.3, 129.9, 119.6, 53.8, 41.1, 33.1, 32.9, 31.0, 30.8, 30.7, 30.6, 30.4, 27.7, 27.6, 26.0, 23.7, 23.7, 19.3, 14.7, 14.6, 13.0; HRMS (FTICR-MALDI) *m/z* calcd. for $C_{46}H_{77}NS_4Si_2$ [M]$^+$ 827.44719; found 827.44559 (δm/m=1.93 ppm).

9-(2-Hexyldecyl)-9H-thieno[2',3':4,5]thieno[3,2-*b*]thieno[2',3':4,5]thieno[2,3-*d*]pyrrole (5a). To a solution of SN5’ **4a:** (488 mg, 0.59 mmol) in THF (57 mL), TBAF (2.97 g, 9.42 mmol) was added. After stirring the mixture at room temperature overnight, the crude product was filtered through a short column (silica gel, PE). This yielded SN5’ **5a** (302 mg, 0.59 mmol, 99%) as

a colorless oil. ^{1}H-NMR (400 MHz, [D$_8$]THF, 20 °C): δ=7.41 (d, ^{3}J=5.2 Hz, 2H, TT-α-*H*), 7.34 (d, ^{3}J=5.2 Hz, 2H, TT-β-*H*), 4.31 (d, ^{3}J(H,H) = 7.9 Hz, 2H, N-C*H*$_2$), 2.16-2.27 (m, 1H, N-CH$_2$-C*H*), 1.14-1.50 (m, 24H, C*H*$_2$), 0.78-0.92 (m, 6H, CH$_2$-C*H*$_3$, CH$_2$-C*H*$_3$) ppm. ^{13}C-NMR (100 MHz, [D$_8$]THF, 20 °C): δ=139.8, 136.1, 124.9, 124.6, 122.2, 118.8, 53.9, 41.1, 33.0, 32.9, 32.9, 32.8, 31.0, 30.8, 30.7, 30.5, 30.4, 27.6, 23.7, 23.6, 14.6, 14.6 ppm. HRMS (FTICR-MALDI): *m/z* calcd. for $C_{28}H_{37}NS_4$ [M]$^+$ 515.18034; found 515.17964 (δm/m=1.36 ppm).

9-(2-Hexyldecyl)-9H-thieno[2',3':4,5]thieno[3,2-b]thieno[2',3':4,5]thieno[2,3-*d*]pyrrole-2,7-dicarbaldehyde (6a): To an ice-cooled and stirred mixture of dry DMF (0.54 mL, 6.98 mmol) and DCE (5 mL) under argon, phosphoryl chloride (0.65 mL, 6.98 mmol) was added dropwise. After stirring the mixture for 3 hrs at room temperature, a solution of SN5′ **5a** (120 mg, 0.23 mmol) in DCE (20 mL) was added dropwise. The resulting mixture was stirred for 2 days at 80 °C and was then hydrolyzed with a saturated $NaHCO_3$ solution and DCM (100 mL) at room temperature for 3 days. The resulting mixture was extracted with DCM (4x100 mL), the combined organic layers were dried over Na_2SO_4, and the solvents were removed under reduced pressure. The crude product was purified by column chromatography (silica gel, DCM) which afforded dialdehyde **6a** as an orange solid (122 mg, 0.21 mmol, 92%). The product was further purified by recrystallization from methanol. M.p. 133.2 °C (DSC). ^{1}H-NMR (400 MHz, [D$_2$]DCM, 20 °C): δ=9.91 (s, 2H, C*H*O), 7.95 (s, 2H, TT-β-*H*), 4.22 (d, ^{3}J=8.0 Hz, 2H, N-C*H*$_2$), 2.04-2.16 (m, 1H, N-CH$_2$-C*H*), 1.10-1.62 (m, 24H, C*H*$_2$), 0.76-0.90 (m, 6H, CH$_2$-C*H*$_3$, CH$_2$-C*H*$_3$) ppm. ^{13}C-NMR (100 MHz, [D$_2$]DCM, 20 °C): δ=183.0, 143.6, 140.4, 137.3, 131.1, 130.1, 123.0, 40.5, 32.4, 32.2, 32.2, 32.2, 32.1, 30.3, 30.0, 29.9, 29.8, 26.9, 23.2, 23.2, 23.1, 14.4, 14.4 ppm. HRMS (FTICR-MALDI): *m/z* calcd. for $C_{30}H_{37}NO_2S_4$ [M]$^+$ 571.17016; found 571.16939 (δm/m=1.35 ppm).

2,2'-{[9-(2-Hexyldecyl)-9H-thieno[2',3':4,5]thieno[3,2-*b*]thieno[2',3':4,5]thieno[2,3-*d*]pyrrole-2,7-diyl)bis(methaneylylidene]}dimalononitrile (7a): 9-(2-Hexyldecyl)thieno[2',3':4,5]thieno[3,2-*b*]thieno[2',3':4,5]thieno[2,3-*d*]pyrrol-2,7-dicarbaldehyd **6a** (122 mg, 0.21 mmol), malononitrile (1.13 g, 17.1 mmol), and ammomium acetate (1.32 g, 17.1 mmol) were dissolved in 1,2-dichloroethane (120 mL) and heated to 80 °C for 3 days. During the reaction time a large excess of malonodinitrile and ammonium acetate was subsequently added (240 eq. in total for each). The solvent was removed under reduced pressure and the precipitate was filtered

over silica gel (DCM). The crude product was recrystallized from methanol and heteroacene **7a** (122 mg, 0.18 mmol, 85%) was isolated as a greenish-black solid. M.p. 260.7 °C (DSC). ^{1}H-NMR (400 MHz, [D$_2$]DCM, 20 °C): δ=7.98 (d, ^4J=0.6 Hz, 2H, TT-β-*H*), 7.87 (d, ^4J=0.6 Hz, 2H, vinyl-*H*), 4.29 (d, ^3J= 8.0 Hz, 2H, N-C*H*$_2$), 2.03-2.15 (m, 1H, N-CH$_2$-C*H*), 1.12-1.50 (m, 24H, C*H*$_2$), 0.77-0.89 (m, 6H, CH$_2$-C*H*$_3$, CH$_2$-C*H*$_3$) ppm. ^{13}C-NMR (100 MHz, [D$_2$]DCM, 20 °C): δ=150.8, 142.1, 138.02, 135.8, 132.4, 131.6, 125.0, 115.0, 114.3, 76.2, 41.0, 32.4, 32.4, 32.4, 32.3, 32.1, 30.3, 30.1, 29.8, 29.8, 27.1, 27.1, 23.2, 23.1, 14.5, 14.4 ppm. HRMS (FTICR-MALDI): *m/z* calcd. for $C_{36}H_{37}N_5S_4$ [M]$^+$ 667.19263; found 667.19108 (δm/m=2.32 ppm).

3,6-Dihexyl-9-(2-hexyldecyl)-9H-thieno[2',3':4,5]thieno[3,2-*b*]thieno[2',3':4,5]thieno[2,3-*d*]pyrrole (9a): In an argon filled Schlenk tube, bithienothiophene **8** (500 mg, 0.83 mmol), Pd(dba)$_2$ (47.6 mg, 0.08 mmol), 1,1'-bis(diphenylphosphino)ferrocene (dppf) (183 mg, 0.33 mmol) and NaO^{t}Bu (795 mg, 8.27 mmol) were dissolved in 20 mL of dry toluene and degassed with argon for 30 minutes. 2-Hexyldecylamine **3a** (240 mg, 0.99 mmol) was added, the resulting mixture was degassed for another 20 min and then stirred at 110 °C for 16 hrs. Afterwards, water was added and the mixture was extracted with DCM. The combined organic layers were washed with a saturated NaCl solution, dried over anhydrous $MgSO_4$, and the solvents were removed under reduced pressure. The crude product was purified by column chromatography (silica gel, PE) which afforded the desired heteroacene **9a** as a yellow liquid (455 mg, 0.67 mmol, 80%). ^{1}H-NMR (400 MHz, CDCl$_3$): δ=6.88 (s, 2H, TT-α-*H*), 4.21 (d, *^3J=7.8 Hz*, 2H, N-C*H*$_2$), 2.74 (t, *^3J=7.8 Hz*, 4H, TT-β-C*H*$_2$), 2.17 (m, 1H, N-CH$_2$-C*H*), 1.79 (q, *^3J=7.5 Hz*, 4H, TT-β-CH$_2$-C*H*$_2$), 1.45 - 1.15 (m, 36H, C*H*$_2$-C*H*$_2$-C*H*$_2$, C*H*$_2$-C*H*$_2$-C*H*$_2$-C*H*$_2$-C*H*$_2$-C*H*$_2$), 0.92-0.80 (m, 12H, C*H*$_3$) ppm. ^{13}C-NMR (101 MHz, CDCl$_3$): δ=139.0, 136.7, 135.5, 123.3, 118.1, 117.1, 40.0, 32.0, 31.9, 31.8, 31.8, 30.0, 29.9, 29.7, 29.6, 29.6, 29.4, 29.3, 28.9, 26.6, 26.6, 22.8, 22.8, 22.7, 14.3, 14.2 ppm. HRMS (FTICR-MALDI): m/z calcd. for $C_{40}H_{61}NS_4$ [M]$^+$ 683.36868; found: 683.36759 (δm/m=1.60 ppm).

9-(4-(*tert*-Butyl)benzyl)-3,6-dihexyl-9H-thieno[2',3':4,5]thieno[3,2-*b*]thieno[2',3':4,5]thieno[2,3-*d*]pyrrole (9b): Under argon, bithienothiophene **8** (500 mg, 0.83 mmol), Pd(dba)$_2$ (47.6 mg, 0.08 mmol), dppf (183 mg, 0.33 mmol), and NaO^{t}Bu (195 mg, 8.27 mmol) were dissolved in degassed toluene (13 mL). The resulting mixture was degassed with argon for 30 min, *p*-*tert*-butylbenzylamine **3b** (0.18 mL, 0.99 mmol) was added and the mixture was degassed for

another 30 min. After stirring the reaction at 110 °C for 24 hrs, the mixture was allowed to cool, the solvent was removed under reduced pressure, and the crude product was purified via column chromatography (silica gel, PE to PE/DCM 4:1) affording SN5' **9b** (450 mg, 0.74 mmol, 90%) as a beige solid. M.p. 140.0 °C-141.6 °C. ^{1}H-NMR (400 MHz, $CDCl_3$): δ=7.35–7.32 (m, 2H, *o*-Ph-*H*), 7.30–7.27 (m, 2H, *m*-Ph-*H*), 6.90 (m, 2H, TT-α-*H*), 5.54 (s, 2H, N-CH_2), 2.73 (t, ^{3}J=7.7 Hz, 4H, TT-β-CH_2), 1.81–1.73 (m, 4H, TT-β-CH_2-CH_2), 1.43–1.31 (m, 12H, CH_2-CH_2-CH_2), 1.25 (s, 9H, CH_3), 0.90 (t, ^{3}J=7.10 Hz, 6H, CH_3) ppm. ^{13}C-NMR (125 MHz, $CDCl_3$, UDEFT): δ=151.3, 139.2, 136.6, 135.3 133.9, 127.4, 126.0, 123.3, 118.4, 117.8, 51.7, 34.7, 31.8, 31.4, 29.6, 29.3, 28.9, 22.8, 15.0 ppm. HR-MS (FTICR-MALDI): m/z calcd. for $C_{35}H_{43}NS_4$ $[M]^+$ 605.22729; found 605.22609 (δm/m=1.98 ppm).

3,6-Dihexyl-9-(2-hexyldecyl)-9*H*-thieno[2',3':4,5]thieno[3,2-*b*]thieno[2',3':4,5]thieno[2,3-*d*]pyrrole-2,7-dicarbaldehyde (10a): In order to prepare the Vilsmeier reagent, a solution of DMF (1.40 mL, 17.6 mmol) in 15 mL DCE was cooled to 0 °C in an ice bath. Phosphoryl chloride (1.70 mL, 17.6 mmol) was slowly added dropwise. After removing the ice bath, the mixture was stirred for 3 hrs at 25 °C. Subsequently, 3,6-dihexyl-9-(2-hexyldecyl)-9*H*-thieno[2', 3':4,5]-thieno[3,2-*b*]thieno-[2',3':4,5]thieno[2,3-*d*]pyrrole **9a** (400 mg, 0.58 mmol) diluted in 35 mL DCE was added to the Vilsmeier reagent and the solution directly turned to deep red. The reaction mixture was stirred at 80 °C for 3 d. In the following, the reaction was hydrolyzed with 200 mL saturated sodium hydrogen carbonate solution and after 5 h additionally with 10 g NaOH. The reaction mixture was stirred for further 3 days. The mixture was poured into 200 mL DCM, the organic phase was washed with saturated NaCl solution for two times, dried over anhydrous $MgSO_4$, filtered, and evaporated. Purification by column chromatography over silica gel (DCM) gave orange solid (375 mg, 0.51 mmol, 88%) as pure product. M.p. 138-139 °C. ^{1}H-NMR (400 MHz, $CDCl_3$, 25 °C) δ=δ 10.04 (s, 2H, C*H*O), 4.09 (d, ^{3}J=7.9 Hz, 2H, N-CH_2), 3.12-3.05 (m, 4H, TT-β-CH_2), 2.06 (m, 1H, N-CH_2-C*H*), 1.89-1.77 (m, 4H, TT-β-CH_2-CH_2), 1.49-1.11 (m, 36H, CH_2-CH_2-CH_2, CH_2-CH_2-CH_2-CH_2-CH_2-CH_2), 0.95-0.77 (m, 12H, CH_3) ppm; ^{13}C-NMR (101 MHz, $CDCl_3$, 25 °C) δ=181.8, 146.8, 141.5, 137.2, 137.1, 129.0, 121.6, 76.9, 53.6, 40.0, 32.0, 31.8, 31.7, 31.7, 30.4, 30.0, 29.7, 29.5, 29.4, 28.2, 26.5, 26.5, 22.8, 22.8, 22.7 14.33, 14.27 ppm. HR-MS (MALDI-FTICR): *m/z* calcd. for $C_{42}H_{61}NO_2S_4$ $[M]^+$ 739.35851; found 739.35735 (δm/m=1.57 ppm).

9-[4-(*tert*-Butyl)benzyl]-3,6-dihexyl-9H-thieno[2',3':4,5]thieno[3,2-*b*]thieno[2',3':4,5]thieno[2,3-*d*]pyrrole-2,7-dicarbaldehyde (10b): To an ice-cooled and stirred mixture of dry DMF (0.19 mL, 2.48 mmol) and DCE (30 mL) under argon, phosphoryl chloride (0.23 mL, 2.48 mmol) was added dropwise. After stirring the mixture for 3 hrs at room temperature, a solution of heteroacene **9b** (50.0 mg, 0.08 mmol) in DCE (6 mL) was added, the resulting red mixture was stirred for 5 days at 70 °C. This was then diluted by DCM (50 mL) and hydrolyzed with a 0.1 M NaOH solution (100 mL) for 24 hours. Afterwards, the phases were separated and the aqueous phase for three times was extracted with DCM. The combined organic phases were washed for three times with water and dried over $MgSO_4$. Column chromatography (silica gel, DCM) of the crude product provided pure dialdehyde **10b** as an orange solid (45.0 mg, 0.07 mmol, 82%). M.p. 189.7-191.1 °C. ^{1}H-NMR (400 MHz, $CDCl_3$): δ=10.05 (s, 2H, C*HO*), 7.35 (d, ^3J=8.4 Hz, 2H, *o*-Ph-*H*) 7.31 (d, ^3J=8.7 Hz, 2H, *m*-Ph-*H*), 5.57 (s, 2H, N-C*H₂*), 3.10 (t, ^3J=7.7 Hz, 4H, TT-β-CH_2), 1.88-1.80 (m, 4H, TT-β-CH_2-CH_2), 1.45-1.31 (m, 12H, CH_2-CH_2-CH_2), 1.26 (s, 9H, CH_3), 0.89 (t, ^3J =7.10 Hz, 6H, CH_3) ppm. ^{13}C-NMR (125 MHz, $CDCl_3$, UDEFT): δ=181.6, 151.9, 146.5, 141.6, 137.2, 136.8, 132.5, 129.0, 127.4, 126.3, 122.1, 51.7, 34.7, 31.6, 31.4, 30.3, 29.4, 28.1, 22.7, 14.2 ppm. HR-MS (FTICR-MALDI): *m/z* calcd. for $C_{37}H_{43}NO_2S_4$ $[M]^+$ 661.21712; found 661.21628 (δm/m=1.37 ppm).

2,2'-[(3,6-Dihexyl-9-(2-hexyldecyl)-9*H*-thieno[2',3':4,5]thieno[3,2-*b*]thieno[2',3':4,5]thieno [2,3-*d*]pyrrole-2,7-diyl)bis(methaneylylidene)]dimalononitrile (11a): 3,6-Dihexyl-9-(2-hexyldecyl)-9*H*-thieno[2',3':4,5]thieno[3,2-*b*]thieno[2',3':4,5]thieno[2,3-*d*]-pyrrole-2,7-dicarbaldehyde **10a** (50.0 mg, 0.07 mmol) was dissolved in 15 mL DCE. Malononitrile (178 mg, 2.70 mmol) and ammonium acetate (208 mg, 2.70 mmol) were added and the reaction was stirred at 80 °C for 2 days. Then, the crude product was precipitated into cold methanol, filtered, and washed with methanol. The pure product **11a** was isolated as a purple solid (54.0 mg, 0.06 mmol, 96%) after column chromatography over silica gel (PE/EA 7:1). M.p. 226-228 °C. ^{1}H-NMR (400 MHz, $CDCl_3$, 25 °C): δ=7.89 (s, 2H, DCV-*H*), 4.14 (d, ^3J=7.6 Hz, 2H, N-CH_2), 2.92 (m, 4H, TT-β-CH_2), 2.03 (m, 1H, N-CH_2-C*H*), 1.76 (m, 4H, TT-β-CH_2-CH_2), 1.41-1.11 (m, 36H, CH_2-CH_2-CH_2, CH_2-CH_2-CH_2-CH_2-CH_2-CH_2), 0.93-0.79 (m, 12H, CH_3) ppm. ^{13}C-NMR (101 MHz, $CDCl_3$, 25 °C): δ=148.5, 147.4, 142.4, 137.5, 130.4, 129.3, 123.4, 115.3, 113.8, 40.4, 31.7, 31.6, 31.3, 31.3, 30.3, 29.7, 29.6, 29.4, 29.2, 29.2, 29.1, 28. 7, 26.4, 22.6, 22.5, 22.4, 14.1, 14.1, 14.0 ppm. HR-MS (FTICR-MALDI): *m/z* calcd. for $C_{48}H_{61}N_5S_4$ $[M]^+$ 835.38098, found 835.38045 (δm/m=0.63 ppm).

2,2'-[(3,6-Dihexyl-9-(2-hexyldecyl)-9*H*-thieno[2',3':4,5]thieno[3,2-*b*]thieno[2',3':4,5]thieno [2,3-*d*]pyrrole-2,7-diyl)bis(methaneylylidene)]bis(1*H*-indene-1,3(2*H*)-dione) (12a): 3,6-Dihexyl-9-(2-hexyldecyl)-9*H*-thieno[2',3':4,5]thieno[3,2-*b*]thieno[2',3':4,5]thieno[2,3-*d*]pyrrole-2, 7-dicarbaldehyde **10a** (150 mg, 0.20 mmol) and 1,3-indandione (118 mg, 0.81 mmol) were dissolved in 50 mL DCE under argon. Piperidine (200 µL, 2.03 mmol) was added and the reaction was stirred at 25 °C for 2 days. The mixture was poured into 50 mL DCM, the organic phase was washed with saturated NaCl-solution for three times, dried over anhydrous $MgSO_4$, filtered, and evaporated. Purification by column chromatography over silica gel (PE/ EA 3:1) and precipitation into cold methanol gave pure product **12a** (190 mg, 0.19 mmol, 94%) as a dark green solid. M.p. 225-227 °C. ^{1}H-NMR (400 MHz, $CDCl_3$, 25 °C): δ=7.85 (m, 4H, Ph-H), 7.72 (s, 2H, vinyl-H), 7.59 (m, 2H, Ph-H), 7.56-7.50 (m, 2H, Ph-H), 4.53-4.37 (m, 2H, 2H, N-C*H*$_2$*)*, 3.00 (t, ^3J=7.8 Hz, 4H, TT-β-C*H*$_2$), 2.16 (m, 1H, N-CH$_2$-C*H*), 1.82-1.67 (m, 4H, TT-β-CH$_2$-C*H*$_2$), 1.55-1.08 (m, 36H, C*H*$_2$-C*H*$_2$-C*H*$_2$, C*H*$_2$-C*H*$_2$-C*H*$_2$-C*H*$_2$-C*H*$_2$-C*H*$_2$), 0.90 (t, ^3J=7.1 Hz, 6H, C*H*$_3$), 0.84-0.70 (m, 6H, C*H*$_3$) ppm. ^{13}C-NMR (101 MHz, $CDCl_3$, 25 °C: δ=190.3, 189.3, 149.7, 142.7, 142.1, 140.4, 138.2, 134.6, 134.3, 133.0, 132.6, 132.0, 122.8, 122.7, 122.6, 121.6, 32.4, 32.2, 32.0, 31.8, 30.6, 30.34, 30.0, 29.7, 29.6, 29.6, 29.2, 27.2, 22.9, 22.9, 22.8, 14.4, 14.3, 14.3 ppm. HR-MS (FTICR-MALDI): *m/z* calcd. for $C_{60}H_{69}NO_4S_4$ [M]$^+$ 995.41094; found 995.40882 (δm/m=2.13 ppm).

2,2'-{{9-[4-(tert-Butyl)benzyl]-3,6-dihexyl-9H-thieno[2',3':4,5]thieno[3,2-*b*]thieno[2',3':4,5] thieno[2,3-*d*]pyrrole-2,7-diyl}bis(methaneylylidene)}bis[1*H*-indene-1,3(2*H*)-dione] (12b): A solution of dialdehyde **10b** (20.0 mg, 0.03 mmol) and 1,3-indandione (17.7 mg, 0.12 mmol) in DCE (4 mL) was treated with two drops of piperidine. After stirring the resulting mixture for 24 hrs at room temperature, the occurring precipitate was filtered off. The solvent of the liquid phase was removed under reduced pressure, the resulting solid was dissolved in little DCM, precipitated with methanol, filtered off, and purified via column chromatography (silica gel, first: DCM, then DCM/EA 97:3). This procedure afforded heteroacene **12b** as a blue solid (25.0 mg, 0.03 mmol, 90%). M.p. (DSC, onset) 315.4 °C. ^{1}H-NMR (500 MHz, d$_2$-TCE, 80 °C): δ=8.02 (s, 2H, vinyl-*H*), 7.92-7.85 (m, 4H, Ph-*H*), 7.68-7.67 (m, 4H, Ph-*H*), 7.51 (d, ^3J*=8.5 Hz,* 2H, *o*-Ph-*H*), 7.41 (d, ^3J*=8.1 Hz,* 2H, *m*-Ph-*H*), 5.71 (s, 2H, N-C*H*$_2$), 3.04 (t, ^3J*=7.6 Hz*, 4H, TT-β-C*H*$_2$), 1.83-1.74 (m, 4H, TT-β-CH$_2$-C*H*$_2$), 1.56-1.25 (m, 12H, C*H*$_2$-C*H*$_2$-C*H*$_2$), 1.22 (s, 9H, C*H*$_3$), 0.87 (t, ^3J*=7.05 Hz*, 6H, C*H*$_3$) ppm. HR-MS (FTCIR-MALDI): *m/z* calcd. for $C_{55}H_{51}NO_4S_4$ [M]$^+$ 917.26955; found

917.26921 (δm/m=0.37 ppm). Due to low solubility, a ^{13}C-NMR spectrum could not be measured.

2,2'-{{[3,6-Dihexyl-9-(2-hexyldecyl)-9*H*-thieno[2',3':4,5]thieno[3,2-*b*]thieno[2',3':4,5]thieno [2,3-*d*]pyrrole-2,7-diyl]bis(methaneylylidene)}bis(3-oxo-2,3-dihydro-1*H*-indene-2,1-diylidene)}dimalononitrile (13a): 3,6-Dihexyl-9-(2-hexyldecyl)-9*H*-thieno[2',3':4,5]thieno[3,2-*b*]-thieno[2',3':4,5]thieno[2,3-*d*]-pyrrole-2,7-dicarbaldehyde **10a** (70.0 mg, 94.6 µmol) and 2-(3-oxo-2,3-dihydro-1*H*-inden-1-ylidene)malononitrile (92.0 mg, 472 µmol) were dissolved in 20 mL solvent mixture of DCE/EtOH 1:1. The reaction was stirred at 60 °C for 24 h. Then, the crude product was precipitated into cold MeOH, and washed with MeOH. Purification by column chromatography over silica (DCM) and precipitation in cold methanol gave pure product **13a** (85.0 mg, 77.8 µmol mmol, 82%) as a dark green solid. M.p. 264-267 °C. ^{1}H-NMR (400 MHz, $C_2D_2Cl_4$, 80 °C): δ=8.97 (s, 2H, vinyl-*H*), 8.68-8.63 (m, 2H, Ph-*H*), 7.92-7.87 (m, 2H, Ph-*H*), 7.73-7.68 (m, 4H, Ph-*H*), 4.42 (d, ^{3}J =8.4 Hz, 2H, N-C*H₂*), 3.15- 3.08 (m, 4H, TT-β-C*H₂*), 2.27 (m, 1H, N-CH₂-C*H*), 1.87 (m, 4H, TT-β-CH₂-C*H₂*), 1.65-1.18 (m, 36H, C*H₂*-C*H₂*-C*H₂*, C*H₂*-C*H₂*-C*H₂*-C*H₂*-C*H₂*-C*H₂*), 0.97 (t, ^{3}J=7.2 Hz, 6H, C*H₃*), 0.86 (m, 6H, C*H₃*) ppm. ^{13}C-NMR (101 MHz, $C_2D_2Cl_4$, 80 °C): δ=187.5, 160.4, 152.1, 143.6, 139.8, 139.0, 136.8, 134.5, 134.2, 134.0, 133.8, 133.8, 124.8, 124.3, 123.3, 121.0, 115.1, 114.7, 74.0, 68.1, 40.3, 32.3, 32.2, 31.7 31.4, 31.3, 30.4, 29.8, 29.4, 29.2, 29.1, 29.0, 26.7, 22.3, 22.3, 22.2, 13.7, 13.7 ppm. HR-MS (FTICR-MALDI): *m/z* calcd. for $C_{66}H_{69}N_5O_2S_4$ $[M]^+$ 1091.43341, found 1091.43366 (δm/m=0.23 ppm).

2,2'-{{{9-[4-(tert-butyl)benzyl]-3,6-dihexyl-9H-thieno[2',3':4,5]thieno[3,2-*b*]thieno[2',3':4, 5]thieno[2,3-*d*]pyrrole-2,7-diyl}bis(methaneylylidene)}bis(3-oxo-2,3-dihydro-1*H*-indene-2, 1-diylidene)}dimalononitrile (13b): A solution of dialdehyde **10d** (20.0 mg, 0.03 mmol) and 2-(3-oxo-2,3-dihydro-1*H*-inden-1-ylidene)malononitrile (17.7 mg, 0.12 mmol) in a mixture of DCE (3 mL) and ethanol (3 mL) was stirred for 2 days at 60 °C. The resulting precipitate was filtered off. The solvent of the liquid phase was removed under reduced pressure, the resulting solid was dissolved in little DCM, precipitated with methanol, filtered off, and purified via column chromatography (silica gel, first: DCM, then DCM/EA 97:3). This procedure afforded heteroacene **13d** as a greenish blue solid (29.6 mg, 0.03 mmol, 97%). M.p. (DSC, onset): 352.6 °C (decomposition). ^{1}H-NMR (500 MHz, d_2-TCE, 80 °C): δ=8.97 (s, 2H, vinyl-*H*), 8.62-8.60 (m, 2H, Ph-*H*), 7.89-7.87 (m, 2H, Ph-*H*), 7.70-7.65 (m, 4H, Ph-*H*), 7.48 (d, ^{3}J=8.4 Hz, 2H, *o*-Ph-*H*), 7.42

(d, ^3J=8.4 Hz, 2H, *m*-Ph-*H*), 5.69 (s, 2H, N-C*H*$_2$), 3.13-3.00 (m, 4H, TT-β-C*H*$_2$), 1.82-1.75 (m, 4H, TT-β-CH$_2$-C*H*$_2$), 1.47-1.28 (m, 12H, C*H*$_2$-C*H*$_2$-C*H*$_2$), 1.22 (s, 9H, C*H*$_3$), 0.87 (t, ^3J=7.1 Hz, 6H, C*H*$_3$) ppm. HR-MS (FTCIR-MALDI): *m/z* calcd. for $C_{55}H_{51}NO_4S_4$ [M$^+$] 1013.29758; found 1013.29633 (δm/m=1.23 ppm). Due to low solubility, a ^{13}C-NMR spectrum could not be measured.

1,4-Bis[(3,6-dihexyl-9H-thieno[2',3':4,5]thieno[3,2-*b*]thieno[2',3':4,5]thieno [2,3-*d*]pyrrol-9-yl)methyl]benzene (14c): Under argon, bithienothiophene **8** (337 mg, 0.56 mmol), 1,4-phenylenedimethanamine (40.2 mg, 0.30 mmol), Pd(dba)$_2$ (32.1 mg, 0.06 mmol), dppf (183 mg, 0.33 mmol), and NaO^{t}Bu (536 mg, 5.57 mmol) were dissolved in dry and degassed toluene (8.89 mL, 83.6 mmol). The reaction mixture was degassed for 30 min and then stirred at 110 °C for 2 days. Toluene was removed under reduced pressure, the residue was dissolved in DCM and filtered through a pad of silica gel. DCM was removed under reduced pressure and the resulting solid was recrystallized from toluene. Dimer **14c** was obtained as a beige solid (191 mg, 0.19 mmol, 67%). M.p. 197.7-199.5 °C. ^{1}H-NMR (500 MHz, d$_2$-TCE, 100 °C): δ=7.34 (s, 4H, Ph-*H*), 6.75 (s, 4H, TT-α-*H*), 5.45 (s, 4H, N-C*H*$_2$), 2.65 (t, ^3J=7.6 Hz, 8H, TT-β-C*H*$_2$), 1.75-1.69 (m, 8H, TT-β-CH$_2$-C*H*$_2$), 1.39-1.23 (m, 24H, C*H*$_2$-C*H*$_2$-C*H*$_2$), 0.86 (t, ^3J=6.9 Hz, 12H, C*H*$_3$) ppm. HR-MS (FTICR-MALDI): m/z calcd. for $C_{56}H_{64}N_2S_8$ [M$^+$] 1020.28297; found 1020.28181 (δm/m=1.14 ppm). Due to low solubility, a ^{13}C-NMR spectrum could not be measured.

9,9'-[1,4-Phenylenebis(methylene)]bis(3,6-dihexyl-9H-thieno[2',3':4,5]thieno[3,2-*b*]thieno [2',3':4,5]thieno[2,3-*d*]pyrrole-2,7-dicarbaldehyde) (15c): To an ice-cooled and stirred mixture of dry DMF (1.36 mL, 17.6 mmol) and dry DCM (50 mL), phosphoryl chloride (1.65 mL, 17.6 mmol) was added dropwise. After stirring the mixture for 3 hrs at room temperature, a solution of dimer **14c** (48.3 mg, 47.3 µmol) in dry DCM (150 mL) was added. The resulting red mixture was refluxed for 7 days at 60 °C under argon and was then hydrolyzed with a 1 M NaOH solution for 24 hrs. The organic layer was extracted twice with DCM, the combined organic layers were dried over anhydrous $MgSO_4$, and the solvents were removed under reduced pressure. The crude product was purified via column chromatography (silica gel, DCM:EA 95:5) which afforded tetraaldehyde **15c** as a yellow solid (17.7 mg, 15.6 µmol, 33%). M.p. 272.2 °C-277.6 °C. ^{1}H-NMR (400 MHz, CDCl$_3$): δ=10.00 (s, 4H, C*H*O), 7.29 (s, 4H, Ph-*H*), 5.54 (s, 4H, N-C*H*$_2$), 3.08 (t, ^3J=7.7 Hz, 8H, TT-β-C*H*$_2$), 1.85-1.79 (m, 8H, TT-β-CH$_2$-C*H*$_2$), 1.45-1.29 (m, 24H, C*H*$_2$-C*H*$_2$-C*H*$_2$), 0.89 (t, ^3J=7.0 Hz, 12H, C*H*$_3$) ppm. HR-MS (FTCIR-MALDI): *m/z* calcd. for

$C_{60}H_{64}N_2O_4S_8$ [M^+] 1132.26263; found 1132.26052 (δm/m=1.86 ppm). Due to low solubility, a ^{13}C-NMR spectrum could not be measured.

2,2',2'',2'''-{{[1,4-Phenylenebis(methylene)]bis(3,6-dihexyl-9H-thieno[2',3':4,5]thieno[3,2-*b*]thieno[2',3':4,5]thieno[2,3-*d*]pyrrole-9,2,7-triyl)}tetrakis(methaneylylidene)}tetrakis [1*H*-indene-1,3(2*H*)-dione] (16c): A solution of tetraaldehyde **15c** (30.0 mg, 26.5 µmol) and 1,3-indandione (61.9 mg, 423 µmol) in DCM (30 mL) was treated with some drops of piperidine. After stirring the mixture for 2 days at room temperature, the solvent was removed under reduced pressure. The resulting solid was dissolved in little DCM and precipitated with methanol. The precipitate was filtered off and purified by multiple column chromatography (silica gel, DCM/EA 9:1). This afforded dimeric SN5' **16c** as a purple solid (22.4 mg, 13.6 µmol, 51%). M.p. (DSC, onset): 340.24 °C (degradation). ^{1}H-NMR (500 MHz, d_2-TCE, 100 °C): δ=7.87 (s, 4H, C*H*O), 7.64-7.61 (m, 8H, Ph-*H*), 7.48-7.41 (m, 8H, Ph-*H*), 7.34-7.32 (m, 4H, Ph-*H*), 5.22 (s, 4H, N-CH_2), 2.79-2.76 (m, 8H, TT-β-CH_2), 1.72-1.66 (m, 8H, TT-β-CH_2-CH_2), 1.42-1.25 (m, 24H, CH_2-CH_2-CH_2), 0.90-0.88 (m, 12H, CH_3) ppm. HR-MS (FTCIR-MALDI): *m/z* calcd. for $C_{96}H_{80}N_2O_8S_8$ [M^+] 1644.36749; found 1644.36397 (δm/m=2.14 ppm). Due to low solubility, a ^{13}C-NMR spectrum could not be measured.

4. ^{1}H-, ^{13}C-NMR spectra

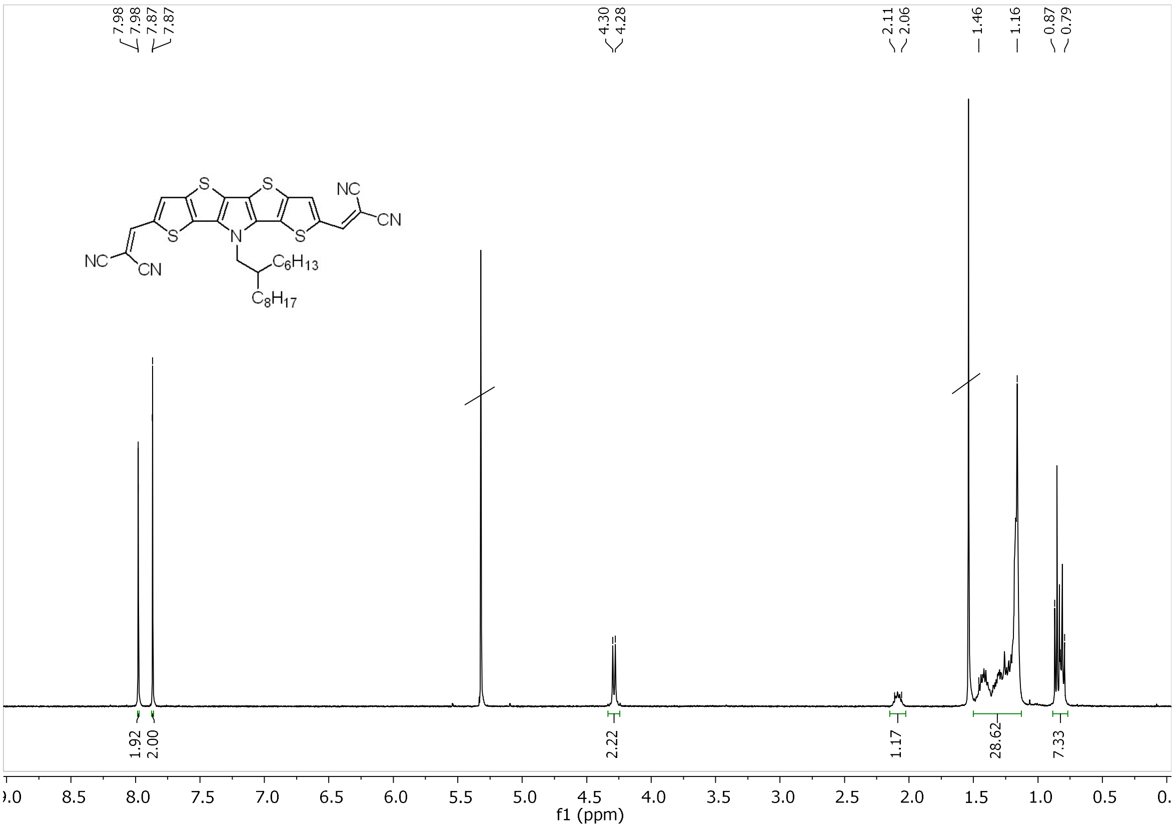

Figure S1a ^{1}H-NMR spectrum of **7a** measured in CD_2Cl_2.

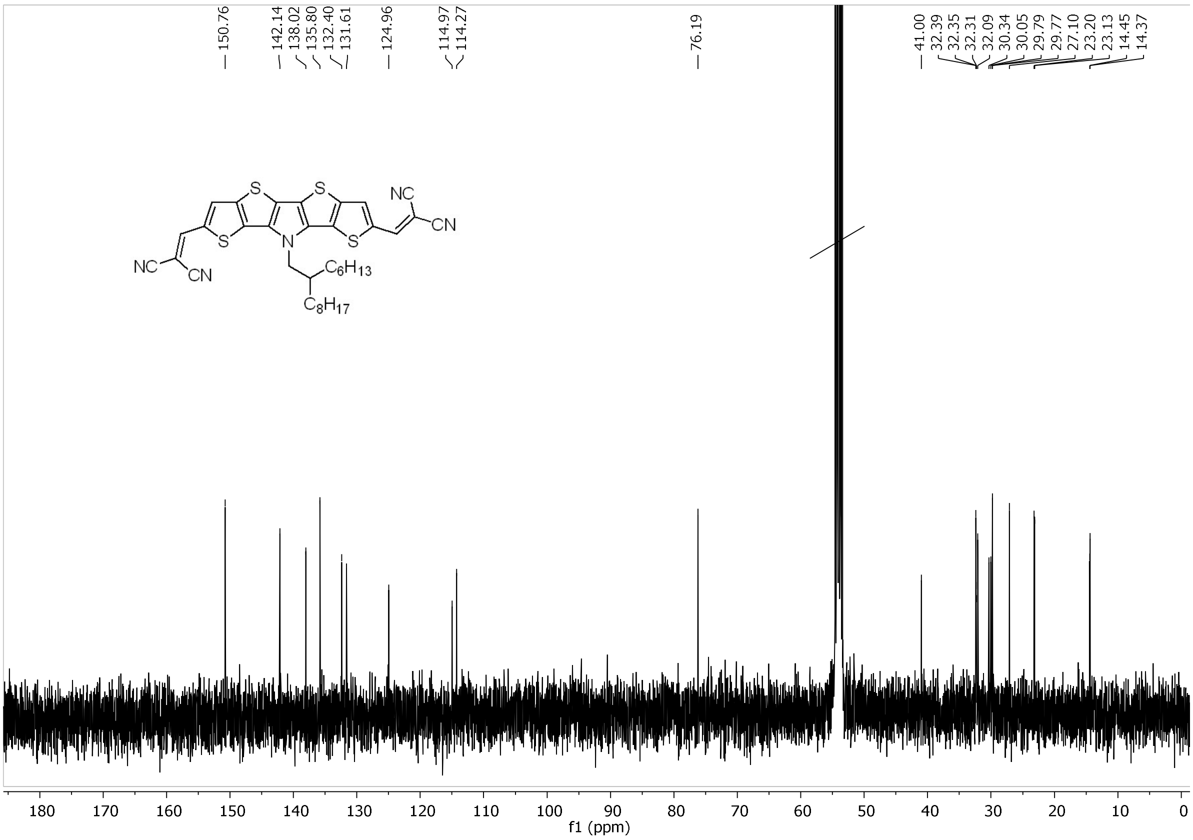

Figure S1b ^{13}C-NMR of spectrum of **7a** measured in CD_2Cl_2.

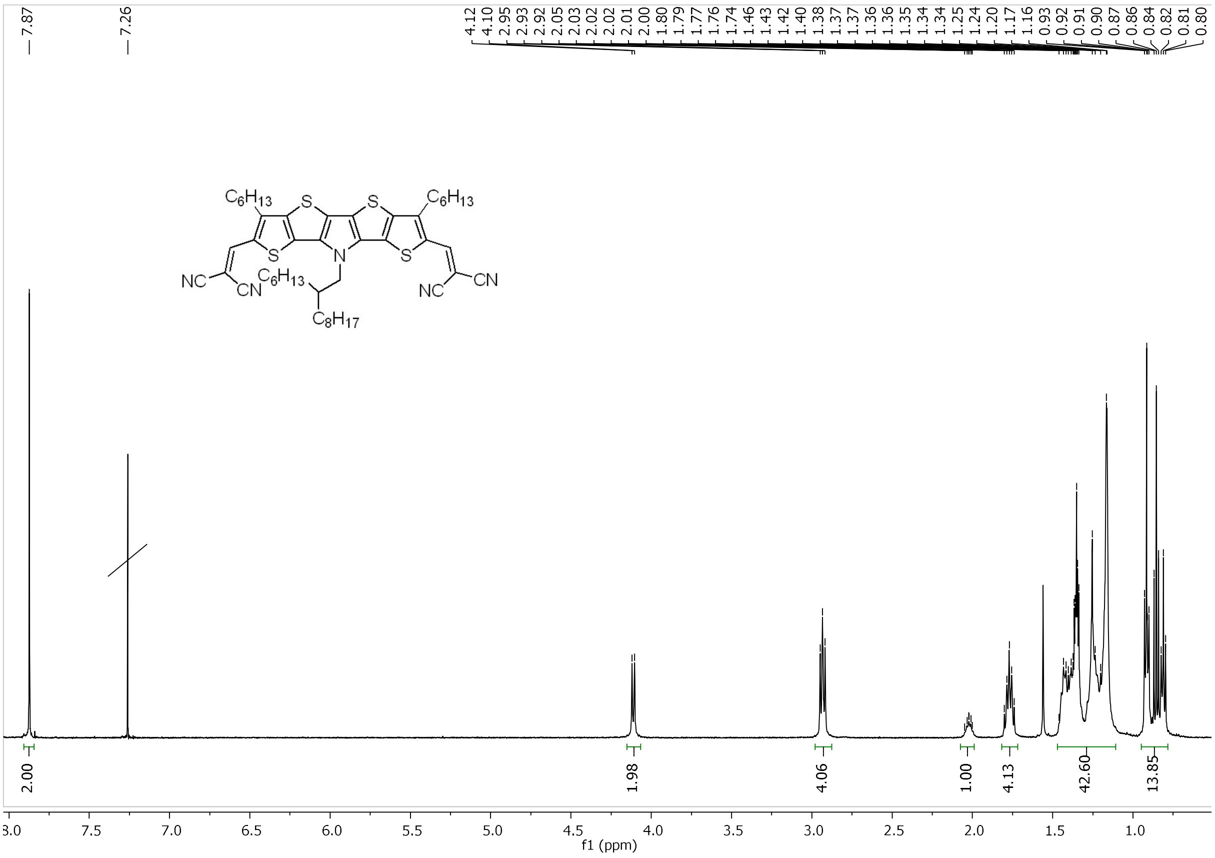

Figure S2a ^{1}H-NMR spectrum of **11a** measured in $CDCl_3$.

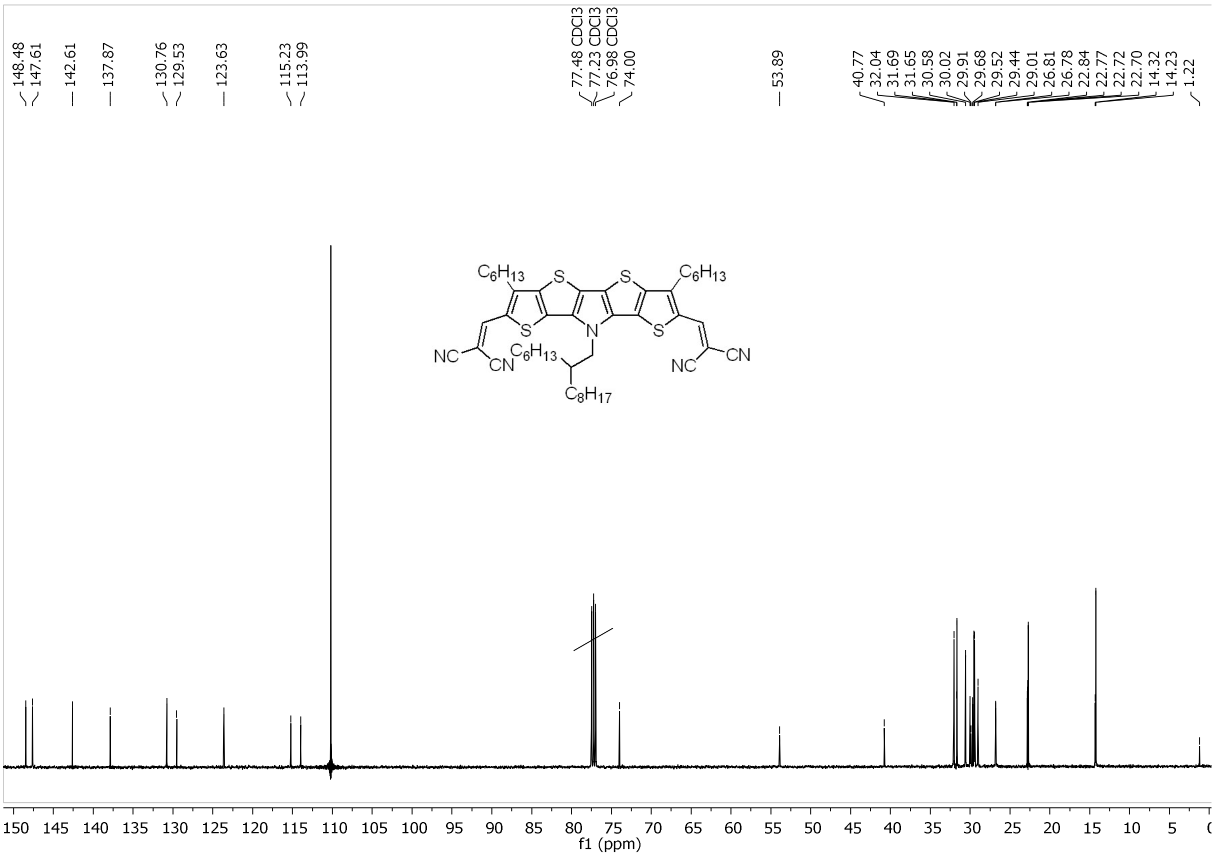

Figure S2b ^{13}C-NMR of spectrum of **11a** measured in $CDCl_3$.

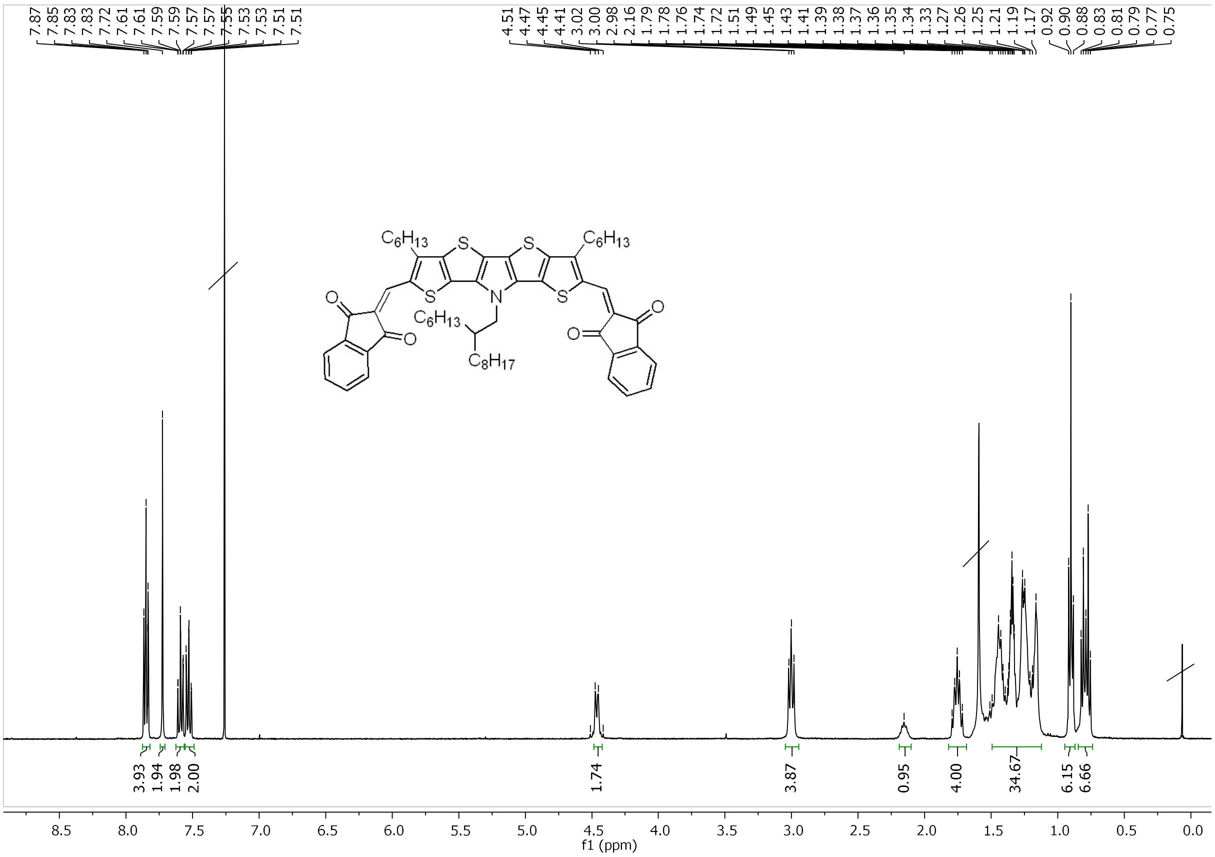

Figure S3a ^{1}H-NMR spectrum of **12a** measured in $CDCl_3$.

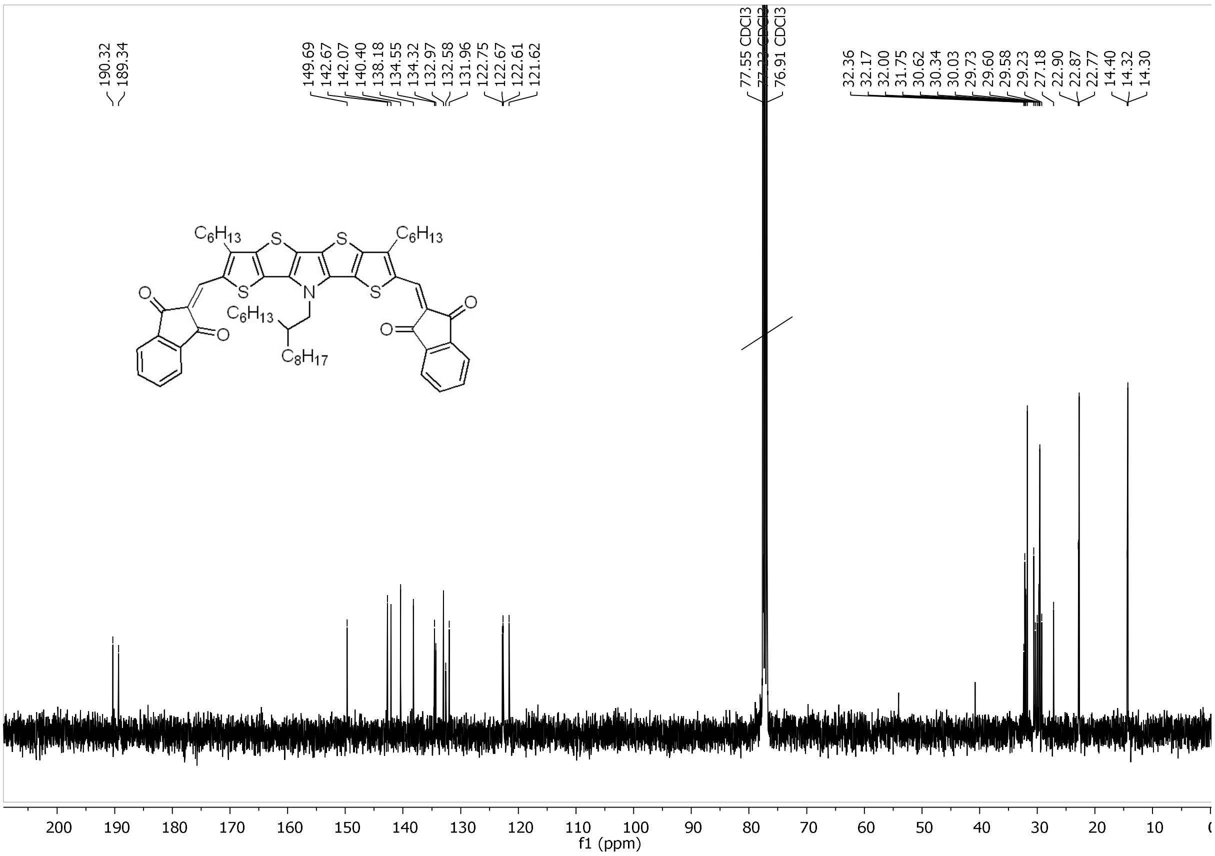

Figure S3b ^{13}C-NMR of spectrum of **12a** measured in $CDCl_3$.

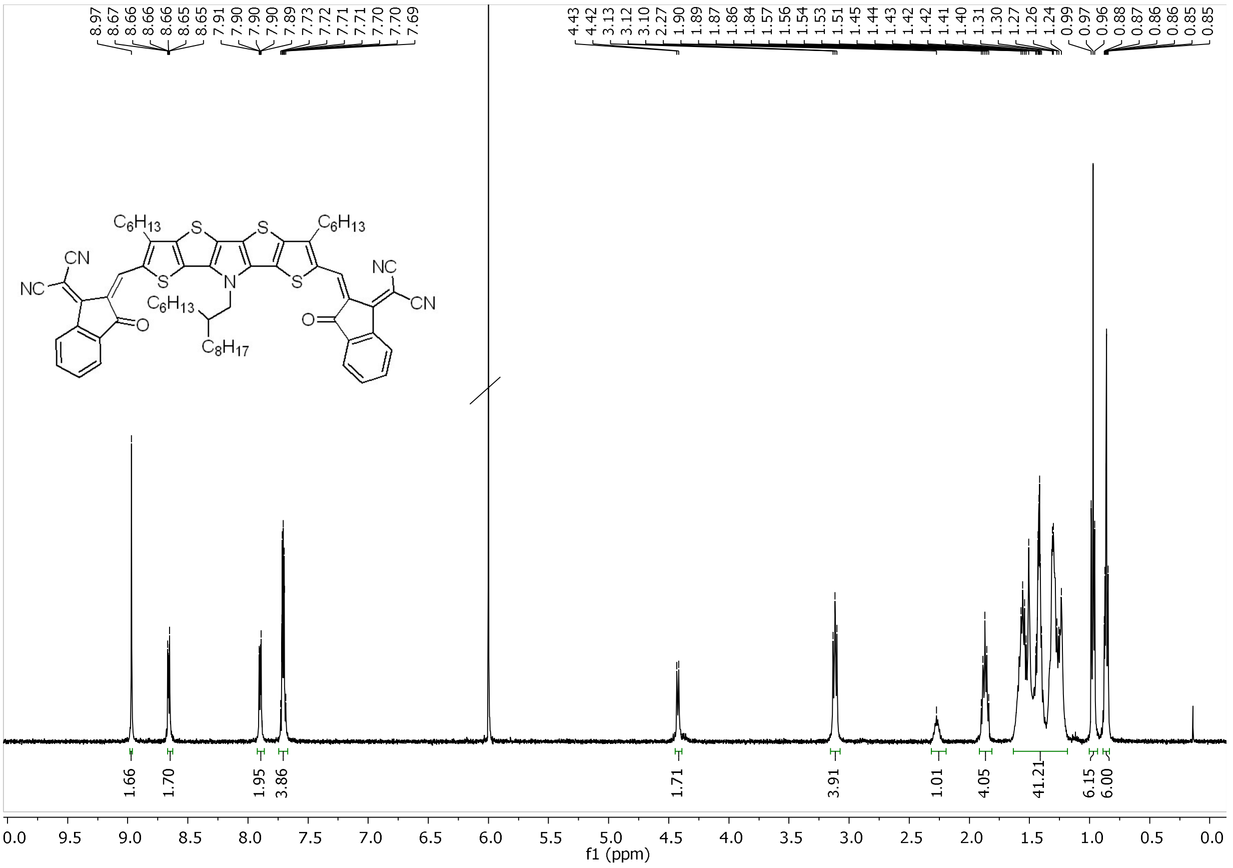

Figure S4a ^{1}H-NMR spectrum of **13a** measured in $C_2D_2Cl_4$.

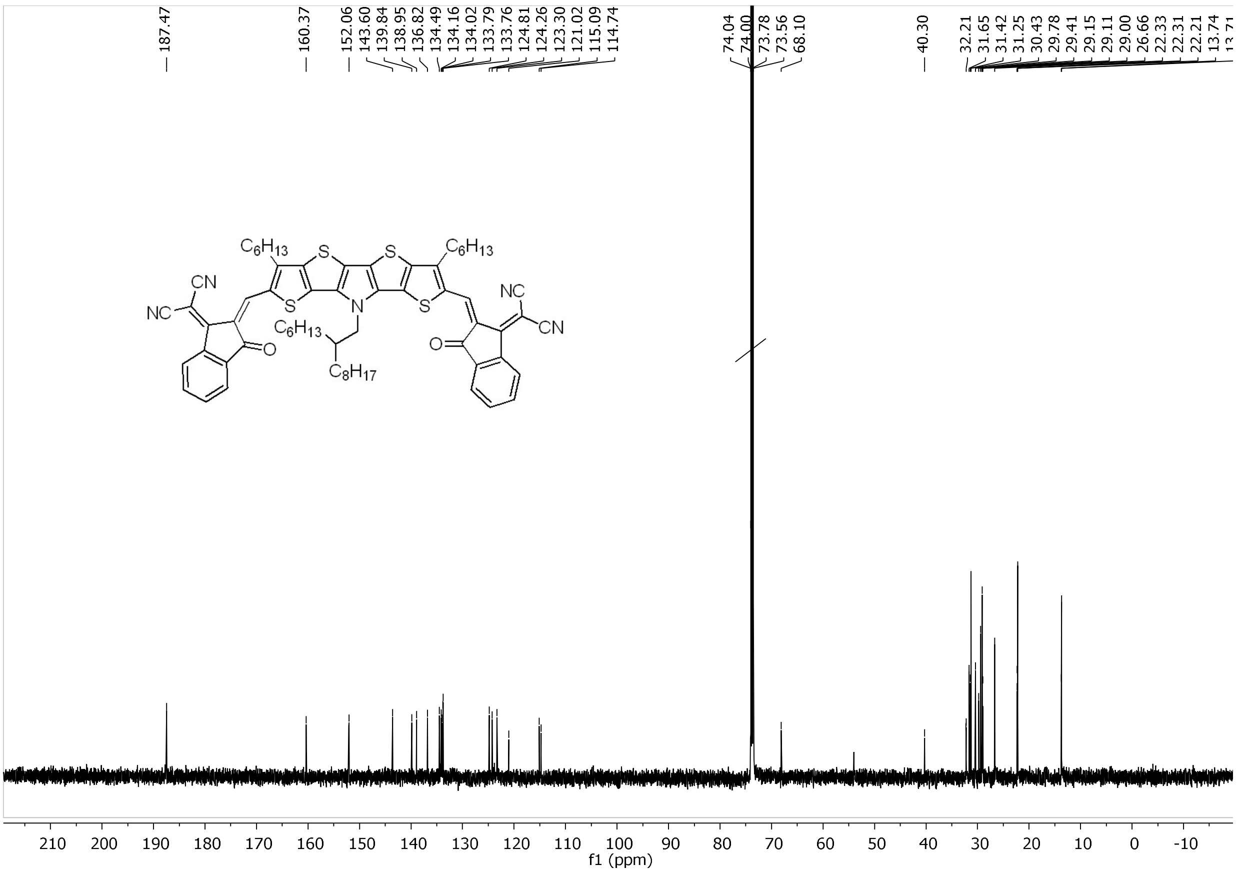

Figure S4b ^{13}C-NMR of spectrum of **13a** measured in $C_2D_2Cl_4$.

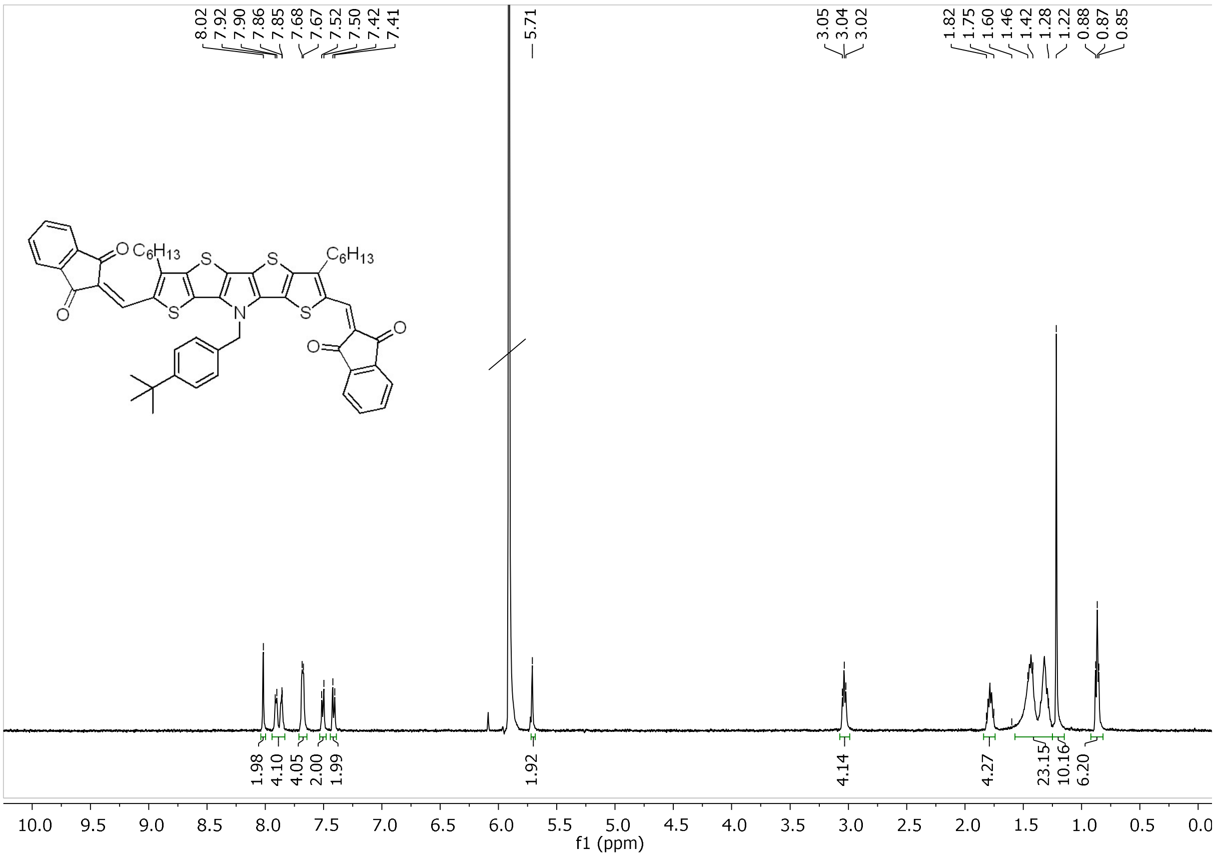

Figure S5a ^{1}H-NMR spectrum of **12b** measured in $C_2D_2Cl_4$.

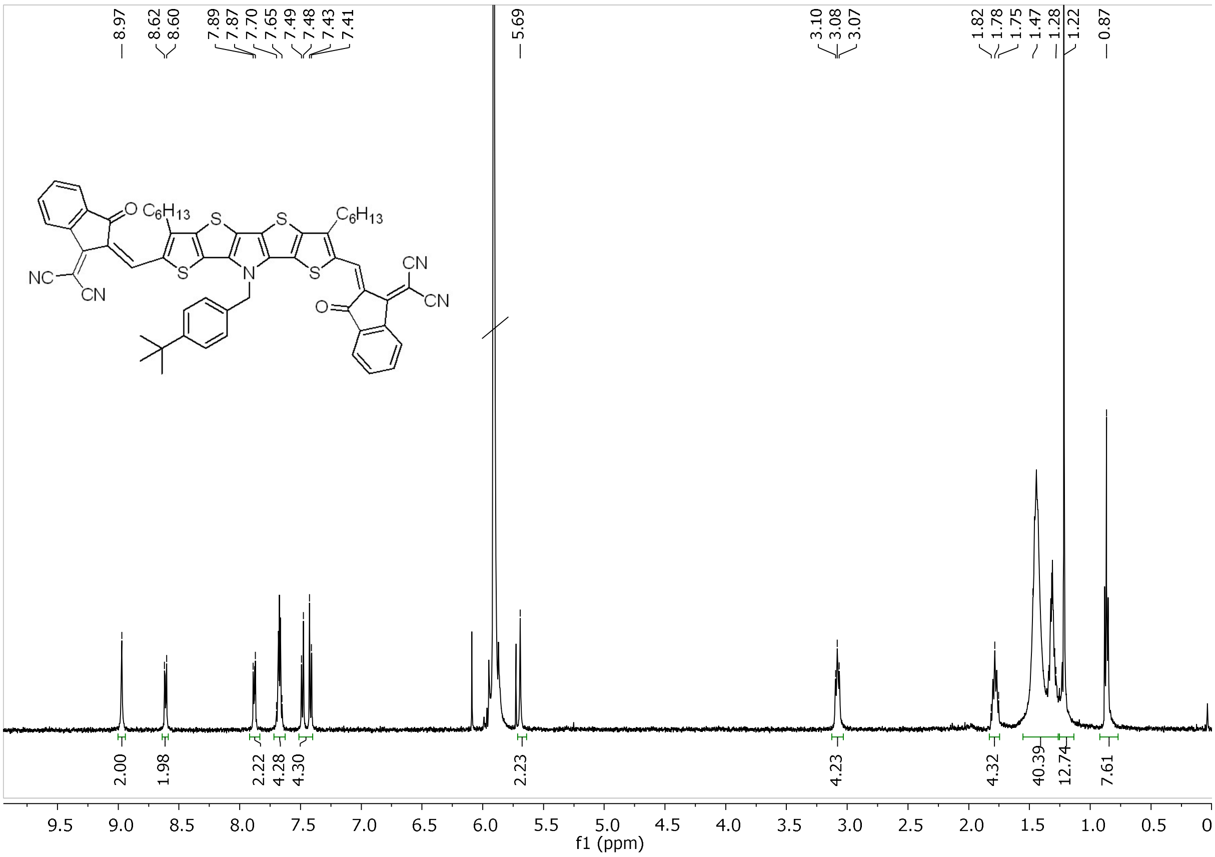

Figure S6a ^{1}H-NMR spectrum of **13b** measured in $C_2D_2Cl_4$.

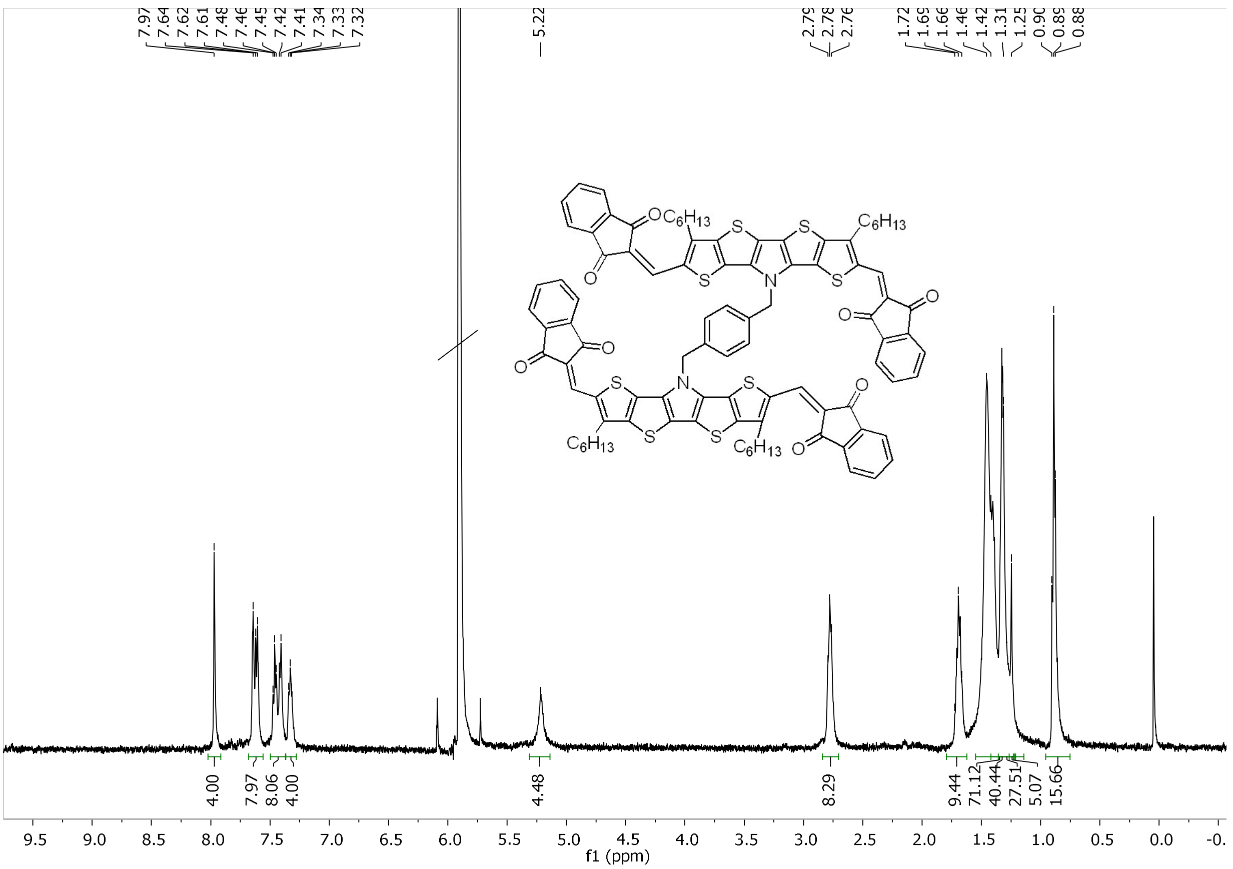

Figure S7a ^{1}H-NMR spectrum of **16c** measured in $C_2D_2Cl_4$.

5. High resolution mass spectra

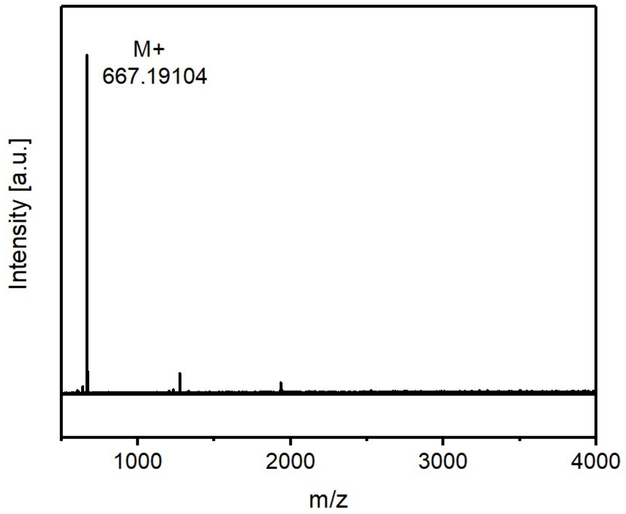

Figure S8 High resolution MALDI FTICR mass spectrum of **7a**.

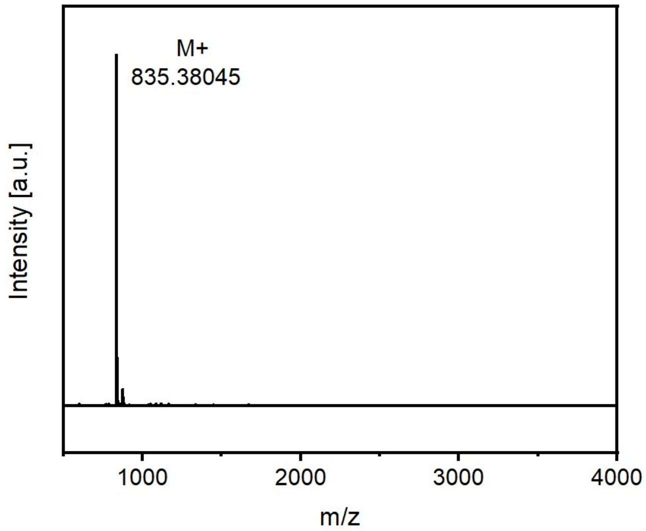

Figure S9 High resolution MALDI FTICR mass spectrum of **11a**.

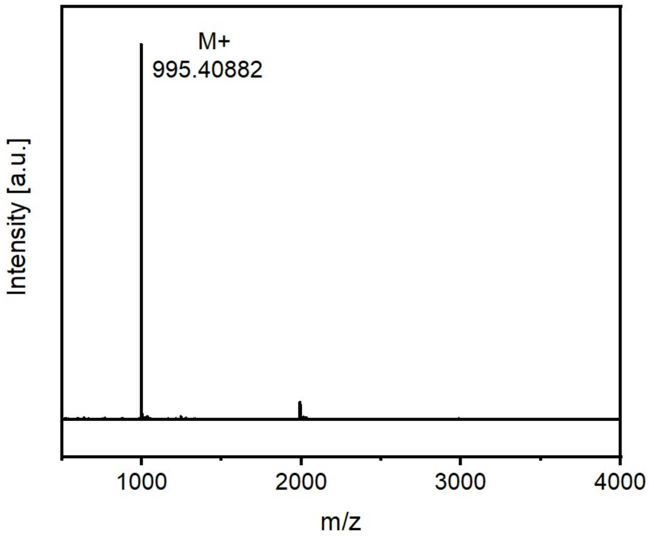

Figure S10 High resolution MALDI FTICR mass spectrum of **12a**.

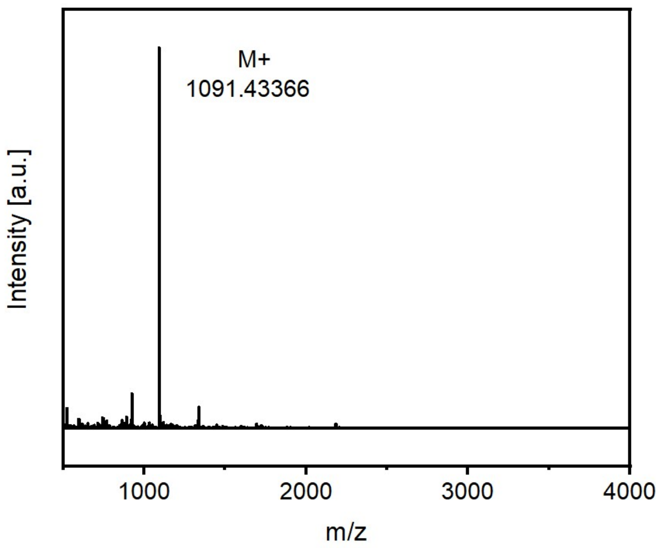

Figure S11 High resolution MALDI FTICR mass spectrum of **13a**.

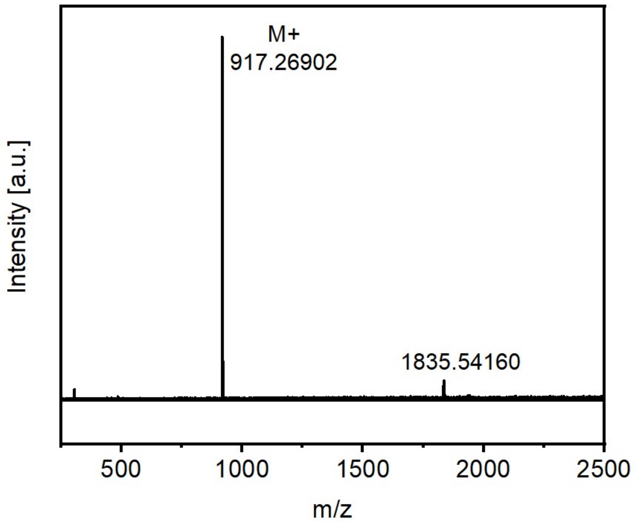

Figure S12 High resolution MALDI FTICR mass spectrum of **12b**.

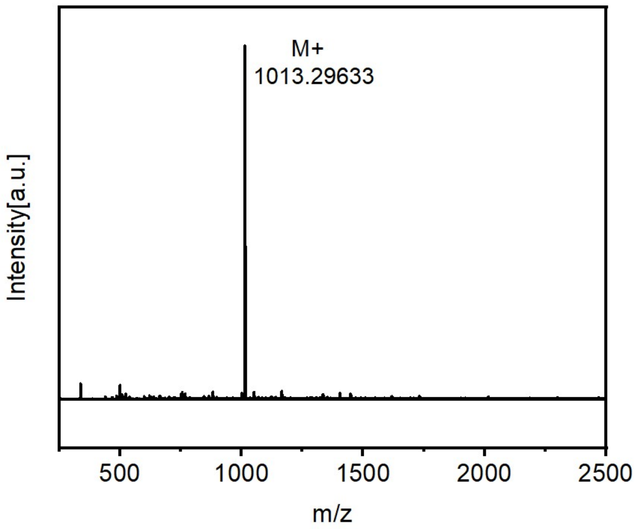

Figure S13 High resolution MALDI FTICR mass spectrum of **13b**.

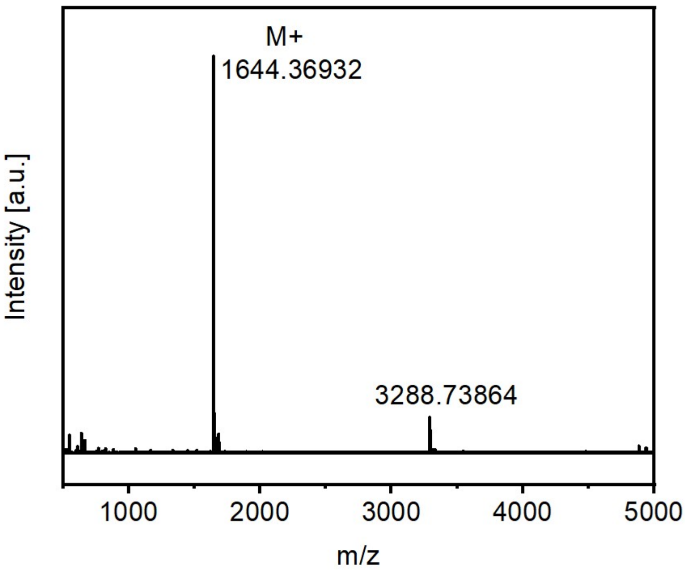

Figure S14 High resolution MALDI FTICR mass spectrum of **16c**.

6. Additional absorption spectra and differential pulse voltammograms

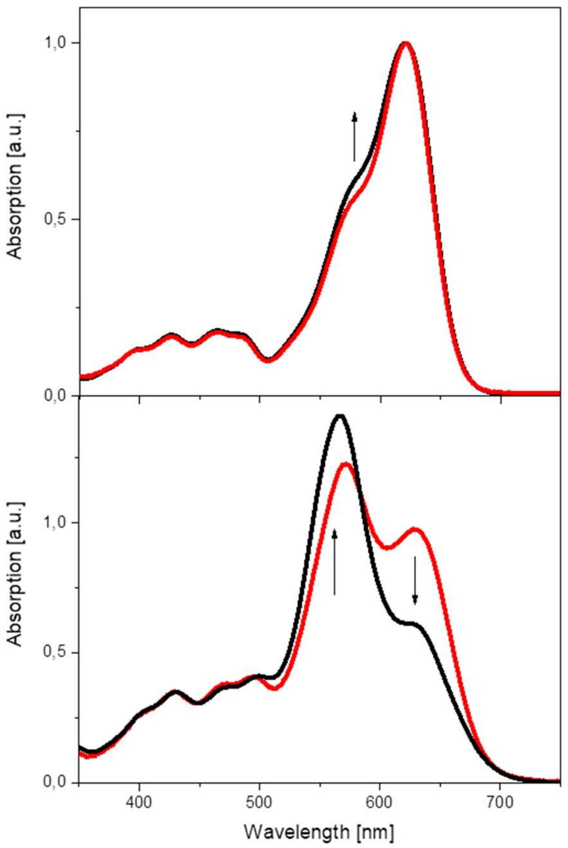

Figure S15. Normalized absorption spectra of SN5'-derivatives **12b** (top) and SN5'-dimer **16c** (bottom) in DCM at low (~ 10^{-7} M, red) and higher (~ 10^{-5} M, black) concentration. Evolution of the bands with increasing concentration are labelled with arrows.

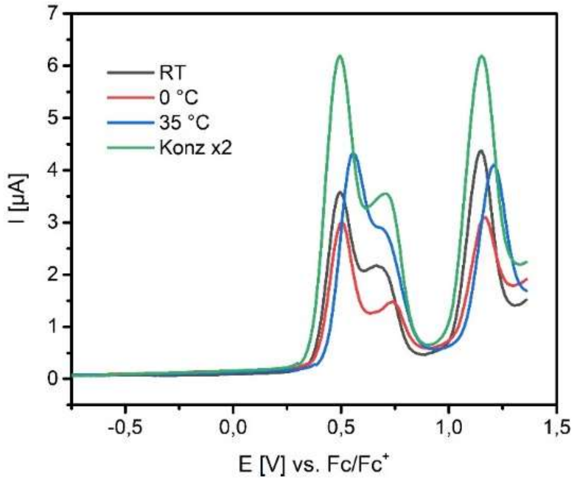

Figure S16. DPV of SN5'-derivatives **12b** at various temperatures and concentration.

7. Organic photovoltaic data

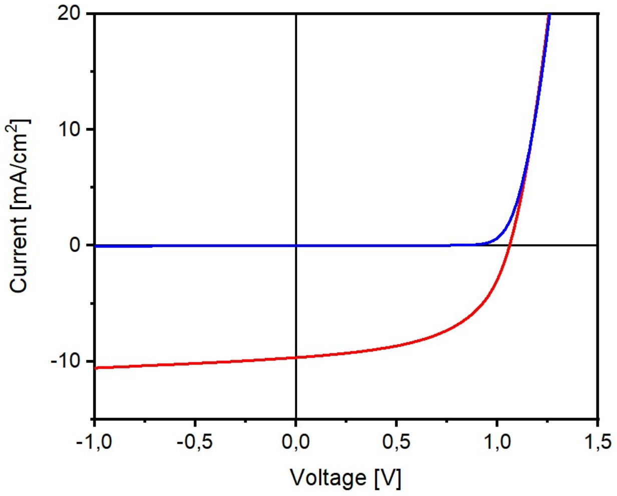

Figure S17. *J-V* curves of optimized BHJ-OSCs of SN5'-derivative **12a** used as donor component under dark (blue) and illumination (red) conditions.

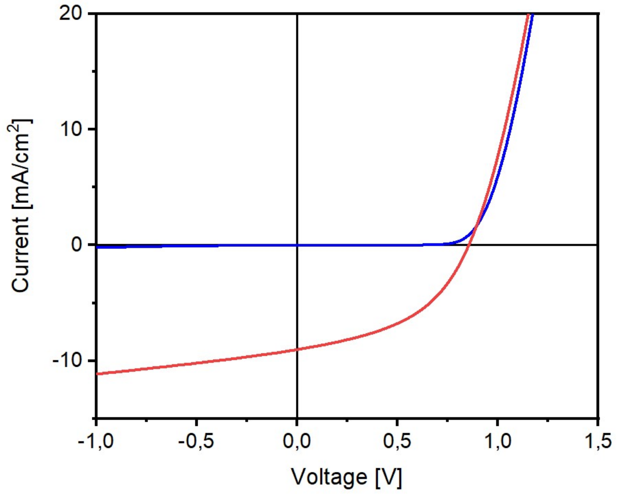

Figure S18. *J-V* curves of optimized BHJ-OSC of SN5'-derivative **13a** used as acceptor component under dark (blue) and illumination (red) conditions.

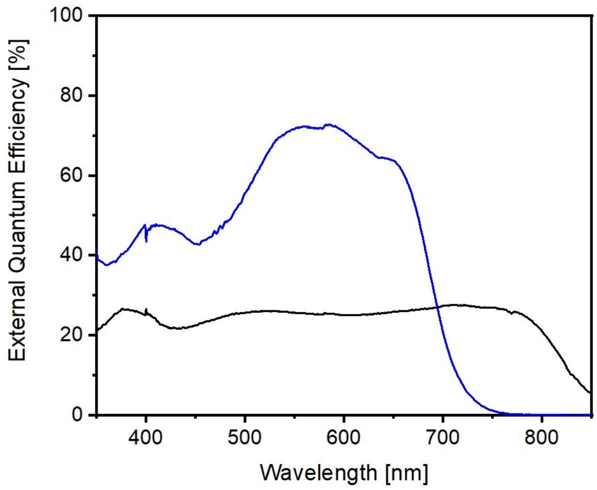

Figure S19. EQE measurements of BHJ-OSCs of **12a**:PCBM (blue) and **13a**:PBDB-T (black).

8. References

[1] C. Wessendorf, A. Perez-Rodriguez, J. Hanisch, A. Arndt, I. Ata, G. Schulz, A. Quintilla, P. Bäuerle, U. Lemmer, P. Wochner, E. Ahlswede, E. Barrena, *J. Mater. Chem. A*, **2016**, *4*, 2571-2580.

[2] C. Wetzel, Dissertation, University of Ulm, **2016**.

[3] C.-L. Chung, H.-C. Chen, Y.-S. Yang, W.-Y. Tung, J.-W. Chen, W.-C. Chen, C.-G. Wu, K.-T. Wong, *ACS Appl. Mater. Interfaces* **2018**, *10*, 6471-6483.

[4] S. Lucas, J. Kammerer, M. Pfannmöller, R. R. Schröder, Y. He, N. Li, C. J. Brabec, T. Leydecker, P. Samorì, T. Marzalek, W. Pisula, E. Mena-Osteritz, P. Bäuerle, *RRL Solar* **2020***,* 2000653.

[5] J. Wan, W. Fang, Z. Li, X. Q. Xiao, Z. Xu, Y. Deng, L. Zhang, J. Jiang, H. Qiu, L. Wu,G. Lai, *Chem. Asian J.,* **2010**, *5*, 2290-2296.

[6] K. A. Bello, L. Cheng, J. Griffiths, *J. Chem. Soc. Perkin Trans*. **1987**, *2*, 815-818.
